# Patient Preferences and Shared Decision Making in the Treatment of Substance Use Disorders: A Systematic Review of the Literature

**DOI:** 10.1371/journal.pone.0145817

**Published:** 2016-01-05

**Authors:** Anke Friedrichs, Maren Spies, Martin Härter, Angela Buchholz

**Affiliations:** Department of Medical Psychology, University Medical Center Hamburg-Eppendorf, Hamburg, Germany; Medical University of Vienna, AUSTRIA

## Abstract

**Background:**

Shared Decision Making (SDM) as means to the involvement of patients in medical decision making is increasingly demanded by treatment guidelines and legislation. Also, matching of patients’ preferences to treatments has been shown to be effective regarding symptom reduction. Despite promising results for patients with substance use disorders (SUD) no systematic evaluation of the literature has been provided. The aim is therefore to give a systematic overview of the literature of patient preferences and SDM in the treatment of patients with SUD.

**Methods:**

An electronic literature search of the databases Medline, Embase, Psyndex and Clinical Trials Register was performed. Variations of the search terms substance use disorders, patient preferences and SDM were used. For data synthesis the populations, interventions and outcomes were summarized and described according to the PRISMA statement. Methodological quality of the included articles was assessed with the Mixed Methods Appraisal Tool.

**Results:**

N = 25 trials were included in this review. These were conducted between 1986 and 2014 with altogether n = 8.729 patients. Two studies found that patients with SUD preferred to be actively involved in treatment decisions. Treatment preferences were assessed in n = 18 studies, where the majority of patients preferred outpatient compared with inpatient treatment. Matching patients to preferences resulted in a reduction on substance use (n = 3 studies), but the majority of studies found no significant effect. Interventions for SDM differed across patient populations and optional therapeutic techniques.

**Discussion:**

Patients with substance use disorders should be involved in medical treatment decisions, as patients with other health conditions. A suitable approach is Shared Decision Making, emphasizing the patients’ preferences. However, due to the heterogeneity of the included studies, results should be interpreted with caution. Further research is needed regarding SDM interventions in patient populations with substance use disorders.

## Background

Despite the increasing relevance of patient involvement in medical decision-making [1–4] as well as its recommendation in treatment guidelines [5], preferences of patients with substance use disorders have rarely been investigated. Patient preferences are to be distinguished in participation and treatment preferences [6]. Participation preference includes a willingness to get involved into the decision-making process: e.g. does a patient want to decide alone or should the physician decide, or both [7]. In contrast, treatment preference includes a preference for one or another treatment or a preference for no treatment at all. Treatment preferences can contain preferences for a setting like inpatient or outpatient treatment, a preference for a specific medication, or a treatment goal. Recent studies showed that patients want to be informed if there are more than one treatment alternatives [8,9]. In a meta-analysis over various health conditions, except patients with substance use disorders, it was proven that patients who were matched to preferred treatments had a higher treatment adherence and improvements in symptom-related outcomes [10] as well as longer retention rates [11].

One method to emphasize patient preferences by a physician during treatment decisions is the model of Shared Decision Making (SDM; [12]), which is to be distinguished from the paternalistic and informed decision-making model. In the paternalistic model the patient has no autonomy in regards to treatment decisions since the clinician decides on what is best for the patient. In contrast in the informed model the clinician has no autonomy since only the patient decides on a treatment option. The responsibility to decide on a treatment within the SDM model lies with both the clinician and the patient [7,13]. Therefore, SDM can be considered a bilateral process that leads to a joint and equivalent treatment decision based on shared information between clinician and patient [14,15]. During the SDM process clinicians contribute evidence-based medical knowledge, experiences and attitudes while patients share their individual perspectives, expectations and goals as well as information regarding needs, values and their daily life. Hence, a decision on a subsequent treatment can be drawn within the framework of evidence-based medicine and individual patient preferences [15,16].

SDM is particularly recommended for so called preference-sensitive medical decisions. Those are situations, where two or more equivalent treatment options are available or consequences of the treatment affect the patients’ daily life. In preference-sensitive decisions, the decision depends largely on the patients’ informed preferences regarding existing treatment options and their individual value of risks and benefits [16]. Hence SDM poses a suitable approach in the treatment of chronic conditions [17] and shows to be accepted [18].

Various decision support tools for SDM have been proven applicable and effective [19]. For clinicians’, information brochures, education and coaching methods have been found important, although no binding conclusion about the most effective tool can be drawn [20]. Examples for patients’ decision support tools are decision aids for e.g. prostate cancer or hepatitis B vaccine.

Those decision aids even have shown a positive effect on patient-practitioner communication, an increase of patients’ involvement as well as an improved knowledge and perception of treatment outcomes [19]. Research has illustrated that clinicians gained more knowledge about the patient, considered more treatment options and were more satisfied with the face-to-face contact if SDM was conducted. Patients were also more satisfied with the clinician-patient contact and with the decision; they gained knowledge about their illness and autonomy with the treatment decision [9]. Although an increase in adherence has been found, research on treatment effects is inconclusive: no effect on depression symptoms with patients with depression [21,22] and no effects on schizophrenia symptoms with patients with schizophrenia [23], but a decrease in psychiatric symptoms and drug consumption [2] could be found for patients with substance use disorders (SUD).

Substance use disorders are considered as chronic conditions where patients have several decisions to make, such as residential versus day hospital treatment [5] (retrieved: http://www.nice.org.uk/guidance/cg115/evidence/cg115-alcohol-dependence-and-harmful-alcohol-use-full-guideline3)). Since treatment options often are equal in regard to their outcome [24] the treatment choice depends on the patients’ individual expectations and preferences. Also the German guideline for screening, diagnosis and treatment of alcohol-related disorders [25] recommends SDM in the treatment of alcohol use disorders. Studies emphasize that a higher degree of involvement in drug treatment leads to more satisfaction with the treatment [26] and a reduction of the severity of drug addiction as well as the severity of other mental health problems [2]. When using a decision aid for patients with risky alcohol consumption a reduction of alcohol consumption can also be shown [27]. Given the promising results of research with regard to other health conditions, we expect that SDM can improve decisional comfort, literacy, patient and health provider communication and even symptom reduction. Existing studies in the field of SUD also show promising results. However, to get an overview regarding the current evidence, a systematic review is needed. Therefore the aim of this study is to give a systematic overview of the literature of patient preferences and Shared Decision Making in the treatment of patients with substance use disorders. Research questions are:

1. To which extent do patients with SUD wish to take part during treatment decisions? (participation preferences)
2. Which aspects within SUD treatment do patients prefer? (treatment preferences)
3. Is treatment more effective, when it matches the patients‘ preferences?
4. Which SDM interventions are available in the treatment of substance use disorders?

## Methods

### Search strategy

A review protocol can be accessed via PROSPERO (http://www.crd.york.ac.uk/PROSPERO/) under the registration code CRD42014009588.

An electronic literature search of the databases Medline, Embase, Psyndex and Clinical Trials Register was performed starting in June 2013. Years 1980 until October 2013 were covered. Further an updated search on Medline was conducted for the time November 2013 until June 2015. The search was run using free text terms as well as Medical Subject Headings (MeSH). Since different databases use different MeSH-terms, the terms were adapted to each database. Therefore, variations of the following search terms were utilized: substance use disorders AND patient preferences OR Shared Decision Making. Search terms can be found in S1 Table.

Additionally hand-searches were accomplished in conference proceedings of the International Shared Decision Making conference (ISDM), the German addiction conferences as well as reference lists of included articles. A sensitive search strategy was applied since it was expected to find only a small number of publications. All searches were transferred to the literature software Mendeley (Mendeley Ltd.) for screening of titles and abstracts of eligible articles. After screening of the titles, duplicates were removed. The remaining full texts were screened by at least one of two independent reviewers (AF and MS). Discrepancies were resolved within discussion with the supervisor (AB).

### Inclusion criteria and data extraction

In this review randomized controlled trials (RCTs), quantitative studies and qualitative studies were included. In case of reviews or meta-analyses, included original articles were evaluated for inclusion in this review. Studies that evaluated interventions with either health care professionals or patients were included in this review. Studies were screened using the following inclusion criteria: 1) substance related disorder, 2) adults, 3) patient preference or 4) Shared Decision Making. Articles were excluded following the criteria of 1) patients with cognitive impairments, 2) children and adolescents, 3) evaluation of patients’ reasons or choices for treatment, and 4) Motivational Interviewing interventions.

Data from the included articles was extracted by using a piloted structured form that was based on the Centre for Reviews and Dissemination Guidelines for Systematic Reviews [28], which can be accessed via http://www.york.ac.uk/inst/crd/SysRev/!SSL!/WebHelp/SysRev3.htm. The data extraction form was framed within PICOS (population, intervention(s), comparator(s), outcomes and study design) and included 1) study identifications, like author, citation and country, 2) study characteristics: the aim and objectives as well as design and inclusion criteria, 3) sample characteristics: study population, patients baseline characteristics, and response rates, 5) results during different time frames. The sample characteristics were differentiated, for intervention and control group. Interventions, baseline and outcome data were extracted again for intervention and if possible control group. This review is reported within the PRISMA guidelines (S2 Table).

### Data analysis

For data synthesis the populations, interventions and outcomes were summarized and described. To compare study characteristics, descriptive statistics were used whereas narratives were used to describe interventions. Similar outcomes were pooled if applicable. *Substance use outcomes* are outcomes directly related to substance use, e.g. reduction of consumption, severity of dependence, or abstinence. Outcomes as health status or psychiatric composite scores were pooled into *mental health outcomes*. *Social-related outcomes* summarize outcomes which are related to social functioning, e.g. family or housing problems or legal assistance. Outcomes like knowledge about treatment options or decisional quality were resumed into *SDM-related outcomes* and aspects like adherence, retention or satisfaction with the treatment were pooled into *process-related outcomes*.

### Quality assessment

Methodological quality of the included articles was assessed with the Mixed Methods Appraisal Tool (MMAT; [29]). The MMAT can be utilized for complex systematic literature reviews without restriction on study type. Therefore the quality of mixed methods, qualitative and quantitative studies can be evaluated. Evaluation takes approximately 14 minutes per study, which is feasible and inter-rater reliability was proven to be moderate to perfect for most of the 19 criteria [30]. Two independent reviewers (AF and MS) conducted the quality assessment and the inter-rater agreement was measured using Cohen’s kappa statistics.

## Results

**Fig 1:**
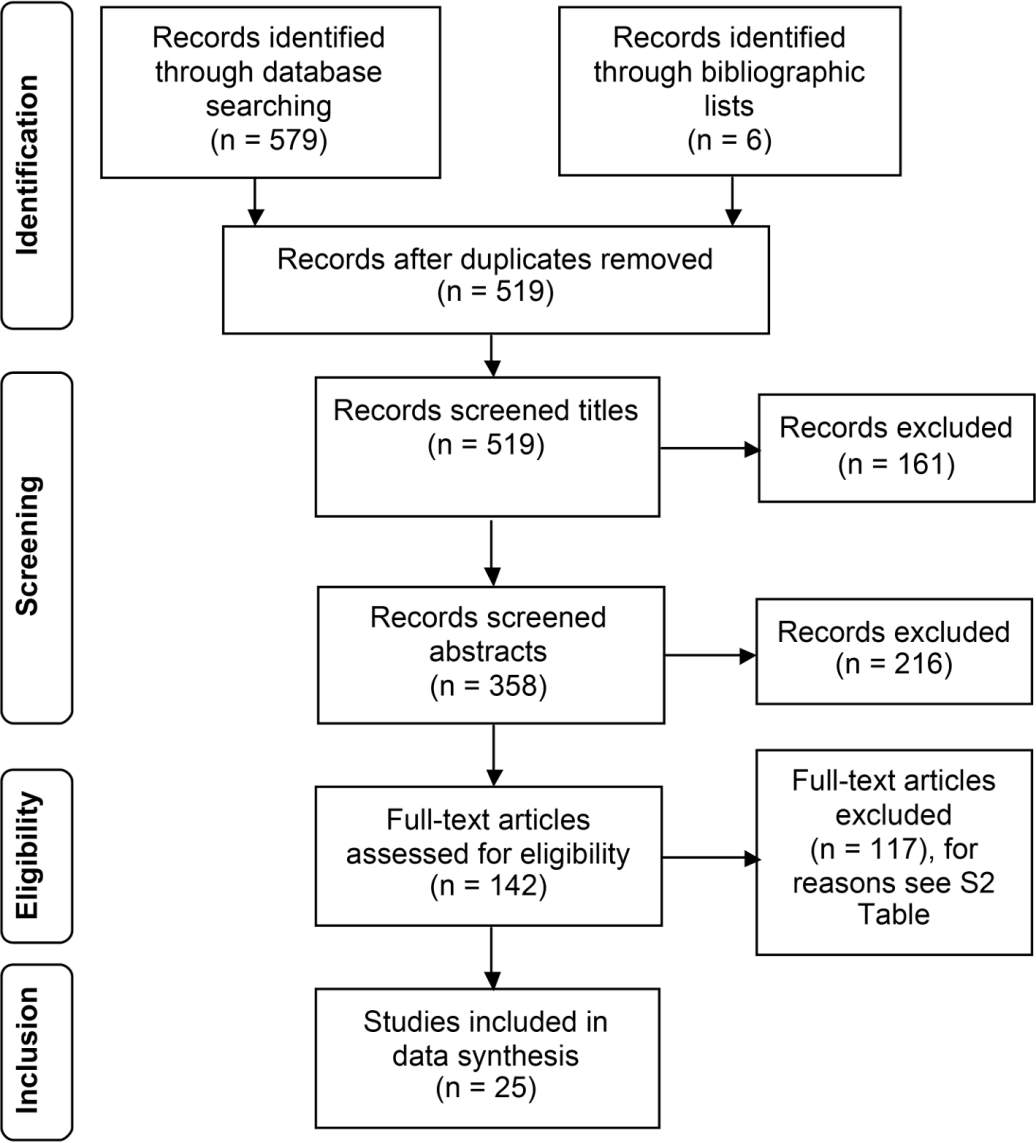
Flowchart of data inclusion.

We identified through database searches N = 579 records and n = 6 records through hand search of included studies. After exclusion of n = 443 references, full text articles were obtained from n = 142 references of which n = 117 were excluded (Fig 1). Reasons of exclusions are summarized in S3 Table. Another n = 3 unpublished articles have been found through conference proceedings and trial registries. We contacted the authors and received a reply from 2 authors saying, data collection is still in process. Altogether N = 25 studies were included in this literature review. Of those studies, n = 10 studies reported only observational data whereas n = 11 studies reported observational and interventional data and n = 7 studies reported only interventional data. N = 4 studies belong to one research group and therefore count only as one study, because of the intervention being the same, as well as author names, settings, and location [31].

### Characteristics of included studies

The majority of studies (n = 15) originated from the United States, whereas n = 3 studies were conducted in the United Kingdom. In Canada, the Netherlands and Germany n = 2 studies have been published respectively and one study originated from New Zealand. A summary of the characteristics of included studies is shown in Table 1. A detailed summary of outcomes can be found in S1 File.

**Table 1:** Characteristics of included studies.

Study & Country	Patient sample & diagnosis	Gender & Mean age	Research objective	Study design
**Participation preferences**				
**Sobell et al. (1992)** * **Canada**^[^^32^^]^	N = 158 problem drinkers	Male: 79,1%, Mean age: 41,0 (11,0) years	Participation preference	Questionnaires
**Neuner et al. (2007)** **** **Germany & Poland**^[^^33^^]^	N = 102 patients in Poland, N = 1009 patients in Germany with alcohol consumption	Male: 70,6% Poland vs. 62,3% Germany, Mean age: 42,7 (17,4) Poland vs. 34,6 (12,8) Germany	Participation preference	Cross-sectional study
**Preference for a service**				
**Liebermann et al. (2014)** ** **USA**^[^^34^^]^	N = 402 problem drinkers	Male: 64.1%; Mean age: 51 (14) years	Treatment preference	Survey
**Preference for a goal**				
**Flach & Diener (2004)** *** **USA**^[^^35^^]^	N = 48 patients with alcohol dependence	Male: 97,8%, Mean age: n.i.	Preference for a treatment goal	Interview, 4-weeks follow-up
**Preference for a medication**				
**White et al. (2007)** ** **United Kingdom**^[^^36^^]^	N = 135 heroin users	Male: 72%, Mean age: 32,2 (7,5) years	Preference for a medication	Survey
**Various preferences**				
**Green (2011)** ** **USA**^[^^37^^]^	N = 218 worried drinkers	Male: 29%, Mean age: 30,61 (11,91) years	Preference for a treatment setting and preference for a therapist	Web-based survey
**Goebert & Nishimura (2011)** ** **USA**^[^^38^^]^	N = 172 clients in residential alcohol treatment	n.i.	Preference for a treatment goal and preference for a treatment service	Interview
**Luty (2004)** *** **United Kingdom**^[^^39^^]^	N = 104 patients with opioid dependence	Male: 62%, Mean age: 34 (0,7) years	Treatment preference and preference for a treatment and preference for a medication	Interviews with card sorting
**Tuten et al. (2007)** ** USA^[^^40^^]^	N = 102 patients with opioid dependence	Male: 66,7%, Mean age: 38 (7,4)	Preference for a treatment setting and preference for a treatment service	Survey
**Dillworth et al. (2009)** ** **USA**^[^^41^^]^	N = 156 adults concerned about their drinking	Male: 39,1%, Mean age: 28.6 (9.0) years	Preference for a treatment goal and treatment preferences	Web-based survey
**Preference matching**				
**Adamson et al. (2005)** ** **New Zealand**^[^^42^^]^	N = 124 patients with mild to moderate alcohol dependence	Male: 57,4%, Mean age: 35,7 (15–59) years	Preference for a treatment service and preference matching	RCT: Matched to preference vs. not matched to preference, 6-months follow-up
**Bernstein et al. (1999)** * **Canada**^[^^43^^]^	N = 99 (n = 55 staff, n = 54 clients) with chemical dependence & Smokers	n.i.	Treatment preferences	Survey, possibility to initiate in smoking program with a choice of goal
**Brown et al. (2002)** * **Canada**^[^^44^^]^	N = 241 patients of whom 71,4% with drug dependence	Male: 67,8%, Mean age: 38,0 (9,3) years	Preference matching	Randomization to Structured Relapse Prevention or 12-Step Facilitation, 3 and 6 months follow-up
**Friedmann et al. (1999)** * **USA**^[^^45^^]^	N = 3255 patients in methadone treatment	Male: 66%, Mean age: 40 or younger	Preference for a treatment service and preference matching	Interview in different treatment settings, 12 months follow-up
**Gossop et al. (1986)** * **United Kingdom**^[^^46^^]^	N = 60 patients of whom 78% with heroin dependence	Male: 75%, Mean age: 26,13 (5,12) years	Preference for a treatment setting and preference matching	Assignment to randomized out- and inpatient, preferred out- inpatient
**Hser et al. (1999)** **** **USA**^[^^47^^]^	N = 171 patients of whom 32% with cocaine as major problem substance	Male: 52%, Mean age: 35 (18–59) years	Treatment preference and preference matching	Interview, 6-months follow-up
Marlowe et al. (2003) ** USA^[^^48^^]^	N = 49 patients with cocaine dependence	Male: 78%, Mean age: 34,29 (7,34) years	Treatment preference and preference matching	Interview & randomization to outpatient or full day cognitive behavioral therapy
**McCrady et al. (2011)** ** **USA**^[^^49^^]^	N = 132 women with alcohol use disorder	Female: 100%, Mean age: 47,6 (9,2) years	Treatment preference	Randomization in self-selected individual counseling group and couples group into one of two treatments
**McKay et al. (1995)** **** **USA**^[^^50^^]^	N = 144 patients of whom 90,3% with alcohol dependence	Male: 100%, Mean age: 41,6 years	Preference for a treatment setting	4 study groups: random day hospital, random inpatient, self-selected day hospital, self-selected inpatient, Follow-up: 3, 6 and 12 months
**McKay et al. (1998)** **** **USA**^[^^51^^]^	N = 171 patients with cocaine dependence	Male: 100%, Mean age: 34,5 years	Preference for a treatment setting	4 study groups: random day hospital, random inpatient, nonrandom day, nonrandom inpatient, Follow-up: 3, 6 and 12 months after rehabilitation
**Sterling et al. (1997)** *** **USA**^[^^52^^]^	N = 127 outpatients with cocaine dependence	Male: 62,6%, Mean age: 32,13 (5,84) years	Treatment preference and preference matching	Interview, self selection of individual therapy or intensive group therapy and randomization into both groups, follow-up: 9 months
**Shared decision-making**				
**a Joosten et al. (2008)** ** **Netherlands**^[^^53^^]^	N = 227 inpatients with either alcohol and/ or drug dependence, N = 34 clinicians, Male: IG 31,3%, CG 44,4%	Male: IG 76,6%, CG 75,9%, Mean age: IG 40,7 (10,3) years, CG 41,2 (11,1) years, Years of working experiences: IG 13,1 (11,6), CG 12,6 (11,5)	Shared Decision Making	RCT with shared decision-making intervention vs. decision-making as usual, Baseline, interim measurement after 8 weeks of treatment (of 3 months inpatient)
**b Joosten et al. (2010)** ** **Netherlands**^[^^54^^]^	N = 111 inpatients with either alcohol and/ or drug dependence	Male: 76,6%, Mean age: 40,7 (10,5) years	Shared Decision Making and preference for a treatment goal	Shared decision-making intervention for patients and clinicians
**c Joosten et al. (2009)** *** **Netherlands**^[^^2^^]^	N = 220 inpatients with either alcohol and/ or drug dependence	Male: IG 73,9%, CG 70,6%, Mean age: IG 40,8 years CG 40,0 years	Shared Decision Making	Shared decision-making intervention vs. decision-making as usual, follow-up: After 8 weeks of treatment (from 3 months), end of treatment and 3-month follow-up
**d Joosten et al. (2011)** ** **Netherlands**^[^^55^^]^	N = 212 inpatients with either alcohol and/ or drug dependence, N = 34 clinicians, Male: IG 31,3%, CG 46,7%	Male: 71,05, Mean age: 42,4 (10,75) years, Years of working experiences: IG 12,3 (11,8), CG 10,1 (9,4)	Shared Decision Making	Randomization by treatment site at clinician level, 3 months follow-up
**Magura et al. (1988)** ^**/**^ **USA**^[^^56^^]^	N = 234 patients in methadone treatment	Male: 73%, Mean age: 32 years	Participative decision making	Evaluation of participative decision making program, 10-months follow-up
**Neumann et al. (2006)** * **Germany**^[^^27^^]^	N = 1139 Patients in emergency department with alcohol related problems	Male: IG 80%, CG 78%, Mean age: IG 30 (24–39) years, CG 31 (25–38) years	Shared Decision Making	Baseline, 6 months follow-up and 12 months follow-up
**Willemsen et al. (2006)** * **Netherlands**^[^^57^^]^	N = 1014 smokers	Male: IG 53,4% CG 54,1%, Mean age 25–54 years: IG 78,8%, CG 78,5%	Shared Decision Making	Decision aid vs. no intervention, follow-up: 2 weeks & 6 months

n.i. = no information given.

/ Met 0% of MMAT criteria

* Met 25% of MMAT criteria

** Met 50% of MMAT criteria

*** Met 75% of MMAT criteria

**** Met 100% of MMAT criteria.

The N = 25 trials were conducted between 1986 and 2014 and included n = 8.729 patients in total. Of these, 56.7% were male patients, ranging from 29 to 100%. In two studies, n = 89 clinicians, e.g. organizational staff [42] or nurses/ social workers [53,55] were interviewed additionally. Participants mean age ranged from 26.13 to 51 years. Of the included studies n = 11 evaluated patients with illicit drug problems, n = 11 of studies had alcohol problems within their scope whereas one study observed patients with either drug or/ and alcohol dependence. Of the included studies, in n = 12 studies, either poly-drug use or psychiatric comorbid disorders were reported.

#### 1) To which extent do patients with SUD wish to take part during treatment decisions? (participation preferences)

Whether patients want to be involved in treatment decisions was evaluated by n = 1 study, investigating the individual selection of treatment goals. Another study investigated the patients’ desire of autonomy during medical decisions (Table 2). Canadian problem drinkers preferred to self-select their treatment goal rather than to let the therapist select a goal. Asking for their generic participation preference, 44% of the patients approved self-selection, 46% preferred shared selection and 11% preferred therapist-selection of treatment goals [32]. German trauma patients who abuse alcohol indicated a higher desire to decide alone than polish patients (m (SD) 16.6 (3.8) vs. 17.7 (4.4) [33].

**Table 2:** Participation preferences.

Research objective	Participation preferences
**Goal selection**	Self selection was preferred to therapist selection (treatment goal)^[^^32^^]^
	Shared selection was preferred to therapist selection (generic selection)^[^^32^^]^
**Desire for autonomy**	German patients had a higher desire for autonomy than polish patients^[^^33^^]^

^[^^32^^]^Sobell et al. (1992), ^[^^33^^]^Neuner et al. (2007).

#### 2) Which aspects within SUD treatment do patients prefer? (treatment preferences)

Treatment preferences were evaluated, ranging from preferences for a setting (n = 8 trials) like inpatient or outpatient treatment, over preference for a treatment modality (n = 12 trials) like Motivational Enhancement Therapy (MET) or general services, e.g. detoxification. For a summary see Table 3 and S1 File.

**Table 3:** Treatment preferences of participants.

Research objective	Treatment preferences
**Preference for a setting**	Professional outpatient was preferred to inpatient^[^^37^^]^
	Residential treatment was preferred to outpatient^[^^39^^]^
	Outpatient treatment was preferred to residential^[^^38^^,^^40^^,^^50^^,^^51^^]^
	Full day treatment was preferred to outpatient^[^^48^^]^
	Strong preference for either in- and outpatient treatment^[^^46^^]^
**Preference for a service**	Motivational Enhancement Therapy was preferred to Non-directive reflective listening^[^^42^^]^
	Alternative Treatment was preferred to Alcohol Anonymous (AA)^[^^41^^]^
	Self-help groups was preferred to online sessions or self-help booklets^[^^37^^]^
	Individual therapy was preferred to couple therapy^[^^49^^]^
	Detoxification was preferred to Narcotic Anonymous (NA)^[^^39^^]^
	AA was preferred to detoxification^[^^38^^]^
	Individual counseling was preferred to AA/NA and group counseling^[^^39^^,^^40^^]^
	Getting help from doctor was preferred to AA^[^^34^^]^
	Individual counseling was preferred to intensive counseling^[^^52^^]^
	An optional smoking cessation program was preferred to no program^[^^43^^]^
	Medical, mental health, family, vocational and housing services were preferred to e.g. communication or anger management services^[^^45^^,^^47^^]^
**Preference for a goal**	Reduction was preferred to no change or abstinence^[^^41^^]^
	Abstinence was preferred to moderate drinking^[^^35^^,^^38^^]^
**Preference for a therapist**	There was no preference for either gender^[^^37^^]^
	There was no preference for sexual orientation of therapist^[^^37^^]^
**Preference for a medication**	Methadone was preferred to Buprenorphine^[^^36^^,^^39^^]^
	Buprenorphine was preferred to Methadone^[^^36^^]^

^[^^34^^]^Lieberman et al. (2014), ^[^^35^^]^Flach & Diener (2004), ^[^^36^^]^White et al. (2007), ^[^^37^^]^Green (2011), ^[^^38^^]^Goebert & Nishimura (2011), ^[^^39^^]^Luty (2004), ^[^^40^^]^Tuten et al. (2007), ^[^^41^^]^Dillworth et al. (2009), ^[^^42^^]^Adamson et al. (2006), ^[^^43^^]^Bernstein et al. (1999), ^[^^45^^]^Friedman et al. (1999), ^[^^46^^]^Gossop et al. (1986), ^[^^47^^]^Hser et al. (1999), ^[^^48^^]^Marlowe et al. (2003), ^[^^49^^]^McCrady et al. (2011), ^[^^50^^]^McKay et al. (1995), ^[^^51^^]^McKay et al. (1998), ^[^^52^^]^Sterling et al. (1997).

Considering treatment preferences, patients with alcohol use disorders rather preferred outpatient / day hospital to inpatient treatment [37,50], although Goebert and Nishimura [38] couldn´t find any differences. However, the majority of opioid [39,40] and cocaine dependent patients [51] preferred outpatient to inpatient treatment. Comparing full day versus outpatient cognitive behavioral therapy, patients with cocaine dependence rather preferred the full-day therapy [48]. Patients with heroin dependence reported having a strong preference, that was independent of the setting, for either out- or inpatient treatment [46].

Preferences for a treatment modality concern either specific treatments e.g. motivational enhancement therapy (MET) versus non-directive reflective listening (NDRL) or general services e.g. detoxification or mental health services. From n = 10 trials, only n = 5 studies reported how patients were informed about different treatments. MET and NDRL was only superficially described to the patients. Dillworth et al. [41] and Sterling et al. [52] gave a written and Mc Crady et al., [49] gave an oral description about treatment modalities. Bernstein and Stoduto [43] offered a 2-hour awareness education session.

When given a superficial indication of aspects of MET (with focus on alcohol consumption and directed by the therapist) and NDRL (with general focus on the patient’s life and less therapist directed), patients with alcohol use disorders preferred MET over NDLR [42]. Comparing alternative treatments, like massages or yoga, versus AA, more patients preferred alternative treatments, after reading information regarding treatment duration, philosophy, treatment goals and format [41] and 29% of worried drinkers preferred self-help support groups compared to online sessions or self-help booklets [37] or receiving help from their physician rather than using internet programs or attending AA [34]. In another study, 84% of women with an alcohol use disorder got a description of modalities and preferred individual rather than couple therapy [49]. Cocaine patients, after gotten a description of treatment plans [52], and opioid dependent patients [39,40] preferred individual counseling to group therapy. Further the majority of chemical dependent smokers as well as staff would support an optional smoking cessation program [43] in addition to the regular treatment, after taking part in a two-hour educational awareness session. Regarding general services, Goebert and Nishimura [38] found significant different preferences between Asian American and Euro American drinkers. The former preferred less detoxification services (p = .013), whereas the latter preferred e. g. significantly less drug abuse programs (p = .013) and more mental health provider services (p = .037). Opioid [40,45] and cocaine patients [47] preferred services for medical, mental health, family, vocational, and housing problems.

Three study groups evaluated the preference for treatment goals of patients with alcohol related problems. Nearly half of the patients preferred a reduction of alcohol consumption to a no problematic amount whereas 15.4% preferred to be completely abstinent [41]. Contradictory, two study groups found that the majority of patients preferred abstinence to moderate drinking [35,50].

Green [37] highlighted that more than half of patients who worried about their drinking habits had no preferences about the gender as well as the sexual orientation of the therapist.

Evaluating preferences for medication with opioid dependent patients, Methadone was preferred, followed by Buprenorphine [39], with the former perceived as having a greater impact on mental health and the latter on heroin use [36].

#### 3) Is treatment more effective, when it matches the patients‘ preferences?

For the comparison of the effectiveness (Table 4, S1 File), outcomes in relation to substance use, to mental health symptoms, to social impact and process aspects were summarized. Outcomes regarding substance use were evaluated as reduction of consumption, severity of dependence, and abstinence as well as scores of the Addiction Severity Index (ASI; [58]).

**Table 4:** Effectiveness of matching patients to their preference.

Research objective	Sig. effect of treatment matched to patients’ preference	No sign. effect
Substance use outcomes**	n = 3^[^^44^^,^^45^^,^^39^^]^	n = 6^[^^42^^,^^43^^,^^46^^,^^47^^,^^50^^,^^51^^]^^*^
Mental health outcomes***	/	n = 2^[^^51^^,^^52^^]^
Social-related outcomes****	/	n = 2^[^^47^^,^^50^^]*^
Process-related*****	n = 1^[^^47^^]^	n = 5^[^^42^^,^^48^^,^^49^^,^^50^^,^^52^^]^

* Due to insufficient information, for [43] and [47], results could not be definitely assigned.

** Substance use outcomes: reduction of consumption, severity of dependence, or abstinence.

*** Mental health outcomes: psychiatric composite (ASI), or health status.

**** Social-related outcomes: family problems, housing problems, or legal assistance.

***** Process-related outcomes: adherence, retention, or satisfaction.

^[^^42^^]^Adamson et al. (2005), ^[^^43^^]^Bernstein et al. (1999), ^[^^44^^]^Brown et al. (2002), ^[^^45^^]^Friedmann et al. (1999), ^[^^46^^]^Gossop et al. (1986), ^[^^47^^]^Hser et al. (1999), ^[^^48^^]^Marlowe et al. (2003), ^[^^49^^]^McCrady et al. (2011), ^[^^50^^]^McKay et al. (1995), ^[^^51^^]^McKay et al. (1998), ^[^^52^^]^Sterling et al. (1997).

From the studies that evaluated matching to patient preferences, four of the studies gave patients the opportunity to choose their preferred treatment. In case they did not have a preference, patients were randomized to one of the offered treatments [46,50–52]. Choosing was permitted in n = 3 studies, from which one study randomized patients [44] and two other studies used observational data [45,47]. In n = 2 studies patients received their preferred treatment [43,49] and in another n = 2 studies preferences were asked prior to randomization but weren’t considered [42,48].

If patients with alcohol use disorders were matched to their preferred treatments, no differences were found for number of drinking days, days intoxicated [50] and reduction of drinking, although matched patients drank trend-wise less heavy than unmatched patients [42]. Patients using illicit drugs tended to have at least trend wise [46,47] or significant better drug-related outcomes, like use in previous 90 days or primary drug use, when they were matched to their preferences [44,45,52]. Although, cocaine using patients showed no significant effects on drug related outcomes, regardless if they were matched to preferences or not [48,51]. Chemical dependent smokers choosing smoking cessation didn´t smoke in the last 7 days whereas patients’ choosing the reduction goal didn´t reduce at all [43].

Patients using illicit drugs didn´t improve in their mental health symptoms if they were matched to their preferences [51,52].

If services were matched to the patients’ needs, drug dependent patients reported a lower problem severity compared to clients with unmatched needs and no needs on different ASI social related severity scores, except legal assistance [47]. Although McKay et al. [51] and Sterling et al. [52] found no effects on those ASI scores for cocaine dependent patients.

If patients with illicit drug use were matched to vocational or housing services, they stayed longer in therapy. Further, if matching occurred for family and medical services, they showed longer retention [47]. Further no differences were found for satisfaction, treatment engagement or retention, for either alcohol [49,50] or illicit drug dependent patients [42,48,51,52].

#### 4) Which SDM interventions are available in the treatment of substance use disorders?

A Shared Decision Making intervention (SDMI) for alcohol or drug dependent patients was evaluated by Joosten et al. [2,53–55].

Prior to the start of the intervention, the clinicians were trained on the use of a SDM protocol, which included aspects of motivational interviewing (MI) as add-on. The latter were used to explore and compare the indicated treatment goals and to reach final agreement on these goals. The SDM intervention contained 5 sessions. In the first session, the clinician introduced the procedure of SDM to the patient and determined the perspectives of patients and clinicians on treatment goals. Cards representing different treatment goals had to be sorted by patient and clinician separately with respect to importance and priority. In the second session, both treatment goals and expectations were explored, compared and discussed. Based on this discussion, a treatment contract was drawn up. In session III an interim evaluation took place (sixth week of treatment). The goals and expectations for treatment were once more explored and discussed, and treatment was adjusted as necessary. At the end of the treatment program, a final evaluation was undertaken. In addition, new goals and expectations were explored. During a three-month follow-up meeting (session V), the goals and expectations agreed upon in the final evaluation were evaluated by the patient only.

In 1988 Magura et al. [56] conducted a study evaluating a decision-making intervention for patients in a Methadone Maintenance Treatment. Team building was used to initiate collaboration between patients and staff, resulting in the establishment of joint patient-staff governance committees. A six-unit, 18-hour team-building curriculum was devised. The training included group discussions and consensual planning of a feasible participative decision-making model as well as the establishment of preliminary objectives.

Applying a computerized tailored decisional support tool for trauma patients with risky alcohol consumption, Neumann et al. [27] administered an individualized feedback to the patients of the intervention group before discharge. It contained computer-generated descriptions of the person’s current drinking status (obtained from the Audit and RTC-Q), compared to safe drinking norms. It further emphasized personal responsibility for determining the need for change and contained advice about the need to develop goals for behavioral change and possible strategies. The feedback was designed to increase motivation, sense of self-efficacy and optimism.

Willemsen et al. [57] evaluated a decision aid for smokers. Participants in the experimental group received a decision aid by post. The decision aid was designed to motivate quitters to use efficacious cessation methods. It contained neutral information on treatment methods available in the Netherlands, distinguishing between efficacious and non-efficacious treatments to help participants deciding on the basis of complete information.

In Table 5 and S1 File outcomes of the different SDM interventions regarding substance use outcomes, psychiatric symptoms, and social outcomes, outcomes in relation to SDM and treatment processes as well as quality of life are described.

**Table 5:** Effectiveness of SDM interventions.

Research objective	Sign. effect of intervention	No sign. effect
Substance use outcomes*	n = 4^[^^2^^,^^27^^,^^54^^,^^57^^]^	n = 4^[^^2^^,^^27^^,^^56^^,^^57^^]^
Mental health outcomes**	n = 1^[^^2^^,^^55^^]^	n = 2^[^^55^^,^^57^^]^
Social-related outcomes***	n = 1^[^^54^^]^	n = 1^[^^2^^]^
SDM-related outcomes****	n = 2^[^^53^^,^^57^^]^	n = 1^[^^53^^]^
Process-related*****	/	n = 2^[^^56^^,^^57^^]^
**Quality of life**	n = 1^[^^2^^]^	/

* Substance use outcomes: reduction of consumption, severity of dependence, or abstinence.

** Mental health outcomes: psychiatric composite (ASI), or health status.

*** Social-related outcomes: family problems, housing problems, or legal assistance.

**** SDM-related outcomes: knowledge of treatments, or decisional quality.

***** Process-related outcomes: adherence, retention, or satisfaction.

^[^^2^^]^Joosten et al. (2009), ^[^^27^^]^Neumann et al. (2006), ^[^^53^^]^Joosten et al. (2008), ^[^^54^^]^Joosten et al. (2010), ^[^^55^^]^Joosten et al. (2011), ^[^^56^^]^Magura et al. (1988), ^[^^57^^]^Willemsen et al. (2006).

### Quality assessment

The methodological quality assessment is shown aggregated in Table 1. A detailed summary of the MMAT results can be found in S4 Table. The inter-rater reliability for the raters was found to be fair with Kappa = .366 (p ≤ .001).

The majority of studies (n = 15) were funded by grants from national institutes for health care research. Two studies were funded by grants from a university, two studies were funded from a public health service provider, one study was funded by the author itself and the funding of another n = 5 studies was not stated.

Because of the comprehensive purpose of this review, no study was excluded owing to its methodological quality.

## Discussion

The aim of this study was to provide a systematic overview about studies which investigated interventions of patient preferences and Shared Decision Making in the treatment of substance use disorders. This is the first study that evaluated this research question. Altogether N = 24 studies could be identified that evaluated either topic using observational or interventional methods.

Regarding participation preferences of the patients, two studies indicated, that patients with alcohol use disorder prefer to be actively involved in the decision-making process [32,33]. Similar results were found in other health conditions, like cancer or cardiovascular conditions or patients seen in general medical encounters [8,9]. Further, patients with acute, like minor trauma, and chronic health conditions, e.g. hypertension, schizophrenia and depression, tended to prefer active involvement but in a shared way with the physician [59,60]. Therefore we strongly support the advice of existing guidelines e.g. NICE [5] or AWMF [25], to involve patients with SUD in treatment decisions.

Regarding treatment preferences, we found that preferences were evaluated for either alcohol or illicit drug populations for different *treatment settings* (e.g. [38,45]) preferring outpatient/ day hospital treatment. Research involving effectiveness of different treatment settings has often methodological problems, but two reviews showed that there is no difference in in- or outpatient treatment for preferences of severe mental ill patients [61] except that the longer the treatment went, the more successful it was for alcohol patients [62]. *Treatment goals* were evaluated comparing abstinence vs. reduction of drinking [35,38,41] with either preference. In terms of effectiveness of controlled drinking, Enggasser et al. [63] showed no different consumption patterns regardless whether patients chose abstinence or controlled drinking.

Regarding the effectiveness of preference-matching, the results pointed in favor for different outcomes related to the substance used for patients with alcohol [42] and illicit drug use disorders [44,45,52]. These results corresponded with the results of a meta-analysis over different health conditions [10,64], indicating varying effect sizes from negligible to large effects, for health related outcomes, although the methodological quality of the included studies was pointed out as a limitation. Concerning the effects on improvement of psychiatric symptoms as well as process-related outcomes, patients with alcohol use disorders, didn´t differ whether they were matched or not [42]. And groups of patients with cocaine dependence showed no differences for psychiatric-, social- and process-related outcomes [48,51,52], yet improvements on psychiatric symptoms and process relations [47]. Looking at patients with depression, it was shown that matching to preferences had a positive effect on process-related outcomes, like completion, working alliance and attendance [65,66]. But for patients with schizophrenia no effect on process-related outcomes was found [58]. Despite these inconclusive results, we can state that treatment matching to patient preferences is a suitable approach, which should be evaluated further taking different methods and study populations and sample sizes into account.

Four study groups evaluated SDM interventions, research question four, with patients with SUD. Apart from the different patient populations for which the interventions were meant they also differed in terms of how extensive they were and whether they included further therapeutic techniques or interventions.

In terms of substance-related outcomes, a significant difference was illustrated for ASI drug score but not for ASI alcohol score [2]. Psychiatric-related outcomes improved significantly, but social-related outcomes didn´t differ between groups. Magura et al. [56] found no differences regarding substance- and process-related outcomes. Neumann et al. [27] and Willemsen et al. [57] illustrated both significant differences for the intervention group and no differences in alcohol- and smoking-related outcomes. SDM-related outcomes were found to be significantly better, which matches the evidence of a Cochrane review regarding other health conditions [67] but process-related outcomes didn´t differ [57]. This result was not unexpected, because similar evidence was shown for other health conditions although most of those studies focused mainly on SDM-related outcomes. Studies with focus on health-related outcomes found inconclusive results as well [21,23]. Because of those promising results, and for the fact that no negative results were found, we conclude that SDM interventions are a suitable method for the treatment of patients with SUD. More research is needed to corroborate the potential of SDM-interventions to improve the substance abuse treatment. For further research, we would suggest to develop and implement decision aids for patients in this context. As Stacey et al. [19] and Willemsen et al. [57] have shown, decision aids have positive effects on symptom related outcomes as well as process related outcomes. Decision aids could be developed regarding treatment goals (abstinence vs. consume reduction) or treatment services (detoxification or AA/ NA or counseling). However, since substance abuse treatment systems differ largely between countries the latter should be developed considering regional or country specific opportunities [68]. Another viable approach could be to train health providers in SDM [68]. But we recommend further research evaluating specific SDM interventions, excluding other treatment approaches but including patient preferences.

## Strengths and Limitations

When interpreting the results of this review, limitations should be considered. Only studies that were published in English or German were included in this review. Systematic reviews are limited by the quality of the included studies as well as their report. Summarizing the methodological quality, it has to be pointed out, that the sample sizes differed from N = 49 to N = 3255 patients with populations with either alcohol-related disorders or drug-related disorders or people with nicotine problems. As control groups, studies included either groups of patients who were randomly assigned or whose preferences were not matched. Therefore, the methodological quality of the evidence was rather insufficient. Although it was not clear whether the studies were only reported not adequately, as it seemed in a few studies. Additionally, there was a possibility of publication bias since no negative outcomes were reported at all. The study selection was not intended to be specific; a sensitive overview was rather aimed with this review. Therefore, the included studies as well as their research questions were heterogeneous. Further they varied in study design, outcome measurements, type of control groups and sample sizes and methodological quality. Hence, results e.g. regarding treatment preferences couldn´t be combined, pooled, compared and generalized adequately. Yet, since there was only limited research on the topic of SDM, all studies were included. Consequently, this literature review enables a first and general overview over relevant aspects. As other strengths, the comprehensive literature search as well as the double rating of the methodological quality can be considered to reduce risk of bias.

## Conclusion

Given the evidence and recommendations of existing guidelines (e.g. Nice guidelines [5] or the German guideline for screening, diagnosis and treatment of alcohol-related disorders [25]) as well as legislation [3,4] and further promising health related results of chronic conditions [23], Shared Decision Making interventions with patients with substance use disorders should be investigated further with putting emphasis on substance-, and social- related outcomes as well as on improvement of psychiatric symptoms. As further aspects of research, we would recommend predictors of patients preferred involvement as well as the consideration of the patients’ severity of addiction when choosing preferred treatments. Moreover, we recommend research to the topic of variations of treatment options in different countries.

## Supporting Information

S1 File Detailed summary of outcomes. (PDF)

S1 Table Search terms. (DOCX)

S2 Table PRISMA Checklist. (DOC)

S3 Table Excluded studies. (DOCX)

S4 Table Results of MMAT. (DOCX)
