# Supplementary material for: Patient Preferences and Shared Decision Making in the Treatment of Substance Use Disorders: A Systematic Review of the Literature
Source: PLoS One. 2016 Jan 5;11(1):e0145817. doi: 10.1371/journal.pone.0145817 (PMC4701396; doi:10.1371/journal.pone.0145817)
Supplement: S1 File — (PDF) [file pone.0145817.s001.pdf]

Participation preferences

Sobell et al. (1992)[32]

		Preference (%)			
		Self	Therapist	No Opinion	Different methods
Q1 (N=158)	“You are just entering treatment here. If given the opportunity, would you prefer to choose your own drinking treatment goal or have the therapist select your goal for you?”	58.1	27.7	14.2	-
Q2 (N=154)	“Which one of the following statements do you most agree with?<br>- Drinking treatment goals should always be assigned by the therapist<br>- All clients should choose their own goals, with therapst advice<br>- Some clients should have their own goals set by the therapist, and others should set their own goals with therapist advice“	43.5	11.0	-	45.5
Q3 (N=137)	“In your case, do you think you would be more likely to achieve your drinking goal if<br>- you selected it yourself<br>- your therapist selected it for you?“	63.5	36.5	-	-

$*p < 0.05$; $**p < 0.01$; $***p < 0.001$

Neuner et al. (2007)[33]

	Decision Making Preference Scale (DMPS) (m ± sd); N=102	
	Warsaw	Berlin
Desire for autonomy	16.6 ± 3.8	17.7 ± 4.4

*$p < 0.05$; **$p < 0.01$; ***$p < 0.001$

Preference for a service

Lieberman et al. (2014)[34]

(%)

		At-risk drinkers* (n=147)	Problem drinkers** (n=65)
Treatment modalities	Getting help from my doctor	61.2	67.7
	Taking a medication that would make it easier to avoid alcohol	44.9	54.8
	Using an internet program	36.1	37.0
	Substance abuse specialty program	31.3	32.3
	Talking to religious or spiritual helper	27.9	24.6
	Alcoholics Anonymous	27.2	23.1
	Taking a medication that would make me sick if I drank	18.4	20.0
	Did not answer	8.8	3.1

** ≥ 5 drinks for men & ≥ 4 drinks for women on ≥ 1 days in the past year*
*** AUDIT score ≥ 8*

Preference for a goal

Flach & Diener (2004)[35]

Vinaigrettes		Ranking (number****)	
Level of cigarette use	Level of alcohol use	T0	T1
Pretreatment amount	Pretreatment amount	9	9
Pretreatment amount	Half of pretreatment amount	6	6
Pretreatment amount	None	3	3
Half of pretreatment amount	Pretreatment amount	7	8
Half of pretreatment amount	Half of pretreatment amount	4	5
Half of pretreatment amount	None	1	2
None	Pretreatment amount	8	7
None	Half of pretreatment amount	5	4
None	None	2	1

****the lowest ranking number is the most preferred vinaigrette

*$p < 0.05$; **$p < 0.01$; ***$p < 0.001$

Preference for a medication

White et al. (2007)[36]

	Preference n (%)		
	Buprenorphine	Methadone	Not sure/neither
Which drug would you recommend to a friend starting treatment?	79 (58.5)	20 (14.8)	36 (26.7)
Which drug gives you a better quality of life	50 (37.0)	35 (25.9)	50 (37.0)
Which would you prefer as substitution therapy?	49 (36.6)	67 (50.0)	18 (13.4)

*$p < 0.05$; **$p < 0.01$; ***$p < 0.001$

Mixed preferences

Green (2011)[37]

Preferences for a setting in %, N= 160	
Professional outpatient:	36
Professional inpatient:	4
Self-help support groups:	29
Computerized treatment:	16
Self-help book:	15

Preference for sexual orientation of therapist in %, N= 164		
No preference for gender:	55	
No preference by sexual orientation:	57	
Men that preferred male therapist:	27	
Women that preferred female therapist:	35	
Heterosexual that preferred heterosexual therapists:	38	
LGB respondents that preferred LGB therapists:	54	(z= 1.7)

*$p < 0.05$; **$p < 0.01$; ***$p < 0.001$

Goebert & Nishimura (2011)[38]

Setting preference	Euro American (N=71)	Native Hawaiian (N=90)	Asian American (N=31)	χ^2
	Preferences for setting (%)			
<u>Social service</u>:				
Social service	47	65	52	5.9
Social worker	22	22	7	5.0
Employee ass. prog.	17	16	19	0.2
Family viol. prog.	11	10	16	0.9
<u>Other</u>:				
Minister	13	12	19	2.4
Traditional healer	7	6	0	2.2

*$p < 0.05$; **$p < 0.01$; ***$p < 0.001$

… continued on the next page

Goebert & Nishimura (2011)[38]

Setting preference	Euro American (N=71)	Native Hawaiian (N=90)	Asian American (N=31)	χ^2
	Preferences for goals or services (%)			
<u>Drinking pattern</u>:				
Abstinence	77	82	61	5.3
Drink moderately	10	18	23	3.3
<u>Skills & education</u>:				
**Relaxation**	**31**	**52**	**58**	**9.4****
**Self-control train.**	**31**	**51**	**48**	**6.2***
**Educational**	**29**	**52**	**52**	**9.6****
Exercise/diet	63	62	45	3.3
Stress manag.	40	49	52	1.8
Social skills train.	37	44	40	0.9
<u>Environmental change</u>:				
**Change environm.**	**32**	**48**	**55**	**6.2***
New friends	51	55	68	2.4
Job change	15	21	27	2.1

… continued on the next page

* $p < 0.05$; ** $p < 0.01$; *** $p < 0.001$

Goebert & Nishimura (2011)[38]

Setting preference	Euro American (N=71)	Native Hawaiian (N=90)	Asian American (N=31)	χ^2
	Preferences for setting (%)			
Allopathie:				
**Marriage couns.**	**9**	**33**	**11**	**13.9*****
Psychotherapy	37	40	52	1.9
Family support	71	83	68	4.2
Prescription medic.	27	18	23	1.6
Alternative/ complementary:				
**Seek minister, priest**	**15**	**36**	**33**	**9.0***
Prayer	46	58	58	2.5
Traditional healer	18	33	28	4.3
Acupuncture	8	17	14	2.4
Herbal medicine	6	10	10	0.8
Hypnosis	5	10	15	2.4

*$p < 0.05$; **$p < 0.01$; ***$p < 0.001$

Luty (2004)[39]

	Initial preference ranking of treatment, N=101	
	n	Score Mean (s.e.)
Oral methadone	101	20.0
Buprenorphine	54	18.3 (4.4)
Rehabilitation	76	18.0 (4.7)
Detoxification	72	18.0 (4.5)
Methadone i.v.	38	17.8 (4.2)
Dihydrocodeine	56	17.5 (3.1)
Diamorphine i.v.	45	17.4 (4.3)
Benzodiazepines	57	17.4 (3.7)
Individual counselling	77	16.8 (4.6)
Group counselling	62	16.5 (4.1)
Lofexidine	25	16.1 (3.5)
Narcotics Anonymous	63	15.5 (4.6)
Acupuncture	49	15.4 (4.8)
Day programme	65	15.3 (3.8)
Naltrexone	14	13.5 (3.1)

*$p < 0.05$; **$p < 0.01$; ***$p < 0.001$

… continued on the next page

Luty (2004)[39]

	Preference ranking (experienced treatment), N=101	
	n	Score Mean (s.e.)
Oral methadone	101	20.0
Buprenorphine	20	19.6 (2.9)
Benzodiazepines	51	18.3 (1.6)
Dihydrocodeine	37	18.2 (2.0)
Individual counselling	50	18.0 (2.3)
Detoxification	33	17.9 (2.9)
Lofexidine	15	17.7 (2.8)
Rehabilitation	24	17.4 (2.7)
Methadone i.v.	18	17.1 (2.5)
Narcotics Anonymous	30	16.8 (2.5)
Diamorphine i.v.	16	16.7 (3.0)
Group counselling	36	16.6 (2.8)
Day programme	27	16.3 (1.7)
Naltrexone	8	14.9 (2.3)
Acupuncture	0	

*$p < 0.05$; **$p < 0.01$; ***$p < 0.001$

Tuten et al. (2007)[40]

		(%)	
	**Setting**		
Aftercare Plans (N=102)	Outpatient	44	
	Recovery housing	26	
	NA meetings	25	
	Inpatient	24	
	“Other“ plans	16	
		**Endorsing**	**Extremely important**
Treatment Services	Individual counseling	73.5	53.7
	Finding employment	66.7	51.5
	Narcotics Anonymous (NA)	61.8	56.7
	Housing	50.0	51.1
	Transportation	50.0	40.6
	Group counseling	49.0	35.8
	Dental treatment	48.0	41.5
	Education	43.1	33.7
	Medical treatment	39.2	44.2
	Social services	39.2	47.3
	Vocational training	37.3	33.7
	Supportive medication	36.3	36.6
	Psychiatric treatment	30.4	36.3
	Relationship counseling	28.4	27.5
	Legal assistance	22.6	23.9

*$p < 0.05$; **$p < 0.01$; ***$p < 0.001$

Dillworth et al. (2009)[41]

Preferences for (%) or (m, sd)

Drinking goal	Reduction to nonproblem level:	50.7		
	No change:	34.0		
	Abstinence if controlled drinking not allowed:	15.4		
		Hetero-sexual	Lesbian, gay or bi-sexual	χ^2
	**Reduction:**	**58.0**	**36.4**	**6.1****
				t
Treatment	Alcoholics Anonymous:	2.24 (1.37)		
	**Alternative treatment:**	**3.09 (1.11)**		**-5.0****
		Hetero-sexual	Lesbian, gay or bi-sexual	t
	Alcoholics Anonymous:	2.3 (1.1)	2.6 (1.7)	1.45
	**Alternative treatment:**	**3.0 (1.1)**	**3.4 (1.1)**	**-2.2***

*$p < 0.05$; **$p < 0.01$; ***$p < 0.001$

Preference-matching

Adamson et al. (2005)[42]

Preference for			Matched	Not matched	χ^2
			(%) or (m, sd)		
4 sessions counselling (n=90)	MET (n=45)	Unequivocal Heavy drinking:	53.3%	59.6%	5.47
	NDRL (n=30)	Change in Global Assessment Score:	2.18 (15.12)	0.78 (14.28)	0.40
	None (n=15)	Satisfaction:	7.84 (3.83)	7.44 (3.35)	0.47
No counselling (n=12)		Perceived Effectiveness:	7.02 (4.28)	6.58 (3.75)	0.44
No preference (n=16)	MET (n=8)	Rapport:	5.87 (5.55)	5.83 (4.78)	0.33
	NDRL (n=6)	Engagement:	5.83 (5.76)	5.47 (4.96)	0.18
	None (n=2)	Attendance:	2.90 (2.81)	3.04 (2.42)	0.09

*$p < 0.05$; **$p < 0.01$; ***$p < 0.001$

Bernstein et al. (1999)[43]

Preference for optional smoking cessation (SC)(n,%)	Patient	Clinician
SC is a good idea:	47 (87.0)	54 (98.2)
Not requiring total abstinence is helpful:	31 (58.5)	29 (53.7)
Allowing clients to smoke is no problem:	31 (75.6)	39 (72.2)
Allowing reduction goals is no problem:	43 (81.1)	31 (56.4)

Engagement in optional smoking cessation (SC)(n,%)	Patient	Clinician
Initiation of contact in CS:	161 (44.1)	10 (55.6)
Joining CS:	172 (38.0)	n.i.****
Abstinence as CS goal:	170 (98.7)	n.i. ****

***** no information given*

… continued on the next page

*$p < 0.05$; **$p < 0.01$; ***$p < 0.001$

Bernstein et al. (1999)[43]

	(n,%)	
	Patients choosing abstinence (n=38)	Patients choosing reduction (n=2)
No smoking in last 7 days:	7 (17.5)	/
Reduction of smoking:	/	0

*$p < 0.05$; **$p < 0.01$; ***$p < 0.001$

Brown et al. (2002)[44]			
	(m, sd) or (days)		
	Matched (n=56)	Not matched (n=51)	F
**ASI drug status at follow-up:**	**0.04 (0.07)**	**0.08 (0.08)**	**4.2***
**Days of substance use in previous 90 days:**	**7.7 (13.6)**	**15.3 (26.6)**	**5.2***

*$p < 0.05$; **$p < 0.01$; ***$p < 0.001$

Friedmann et al. (1999)[45]

	Preferences for a treatment service n (%); N=3255			
	Service needed		Service not needed	
	Matched	Not matched	Matched	Not matched
Medical:	1332 (40.9)	736 (22.6)	683 (21.0)	504 (15.5)
Mental Health:	683 (21.0)	1382 (42.3)	159 (4.9)	1031 (31.7)
Family:	830 (25.5)	1478 (45.4)	246 (7.6)	700 (21.5)
Vocational:	327 (10.0)	1670 (51.4)	129 (4.0)	1127 (34.6)
Housing:	434 (13.3)	1616 (49.4)	85 (2.6)	1129 (34.7)
Mean no. of services:	1.11	2.11	0.40	1.38

	Percent of need service matched r
**Overall drug use improvement:**	**0.054****
**Primary drug use improvement:**	**0.045***

$^{*}p < 0.05$; $^{**}p < 0.01$; $^{***}p < 0.001$

… continued on the next page

Friedmann et al. (1999)[45]

	Effect of matching services parameter estimate (sd)	
	Overall drug use improvement	Primary drug use improvement
Medical:	0.174 (0.091)	0.125 (0.070)
Mental Health:	0.134 (0.088)	0.000 (0.067)
**Family:**	0.162 (0.084)	**0.140 (0.065)***
**Vocational:**	**0.427 (0.121)*****	**0.284 (0.092)****
**Housing:**	**0.472 (0.105)*****	**0.233 (0.081)****

	Effect of matching services over time parameter estimate (sd)	
	Overall drug use improvement	Primary drug use improvement
**Percent of needs matched:**	**0.049 (0.021)***	**0.028 (0.016)**
**Percent of needs matched without duration:**	**0.086 (0.021)*****	**0.050 (0.016)****
**Treatment duration without matched needs:**	**0.025 (0.003)*****	**0.015 (0.002)****

*$p < 0.05$; **$p < 0.01$; ***$p < 0.001$

Gossop et al. (1986)[46]

	(n, %)		
	Preferred group	Random group	χ^2
Successful withdrawal:	40 (58)	20 (35)	2.7
	Inpatient group	Outpatient group	χ^2
**Complete withdrawal:**	**25 (81)**	**5 (17)**	**21.6*******
**Staying in contact to service:**	**15 (29)**	**16 (55)**	**9.19****

*$p < 0.05$; **$p < 0.01$; ***$p < 0.001$

Hser et al. (1999)[47]

Services	(n) or (%)		
	Desired	Received	Received service desired
Vocational:			
Tutoring	63	5	7.9
Job training	116	12	10.3
Practice skills:			
Communication train.	99	14	14.1
Anger management	76	25	32.9
Money management	90	11	12.2
Housing:			
Locate housing	108	11	10.2
Transportation:	116	62	53.4
Legal:			
Legal assistance	84	9	10.7

*$p < 0.05$; **$p < 0.01$; ***$p < 0.001$

… continued on the next page

Hser et al. (1999)[47]			
	(n) or (%)		
Services	Desired	Received	Received service desired
<u>Social</u>:			
Social outings	84	18	21.4
<u>Cultural</u>:			
Religious emphasis	64	25	39.1
Cultural/ ethnic emphasis	30	13	43.3
<u>Family</u>:			
Service for children	65	14	21.5
Children allowed at treatm.	51	18	35.3
Parenting skills training	65	32	49.2

*$p < 0.05$; **$p < 0.01$; ***$p < 0.001$

… continued on the next page

Hser et al. (1999)[47]

Services	Desired	Received	Received service desired
	(n) or (%)		
Medical:			
General medical serv.	107	47	43.9
Pregnancy	28	3	10.7
Medication allowed	90	18	20.0
HIV related:			
HIV testing	85	31	49.7
AIDS counseling	53	25	47.2
Counseling/ self-help:			
Individual counseling	131	114	87.0
Group counseling	102	81	79.4
Family/ couple couns.	87	17	19.5
12-step groups	107	46	43.0

… continued on the next page

*$p < 0.05$; **$p < 0.01$; ***$p < 0.001$

Hser et al. (1999)[47]

Needs/services	Days in treatment (numbers of subjects in this category)		
	Matched	Unmatched	No Needs
**Vocational services:**	**164 (12)***	**99 (104)***	**104 (55)***
**Childcare:**	**156 (18)***	**104 (33)***	**98 (120)***
Housing services:	151 (11)	103 (97)	100 (63)
Parenting skills:	121 (32)	96 (33)	103 (106)
**Transportation:**	**118 (62)***	**81 (54)***	**114 (55)***
Legal assistance:	117 (9)	110 (75)	100 (87)
Family counseling:	106 (17)	94 (70)	114 (84)
Medical services:	94 (47)	115 (60)	104 (64)
Cultural/ ethnic emphasis:	92 (13)	98 (17)	107 (141)

… continued on the next page

*$p < 0.05$; **$p < 0.01$; ***$p < 0.001$

Hser et al. (1999)[47]

Needs/services	Improvement in ASI scores (%)		
	Matched	Unmatched	No Needs
Vocational services:	32	20	14
Housing services:	70	37	25

**Improvements in all ASI severity scores except for legal assistance (not specified in publication)*

Needs/services	Improvement in ASI score drug use (%)		
	Matched	Unmatched	No Needs
Vocational services:	22	36	31
Housing services:	50	23	41
Parenting skills:	45	20	31

**No other improvements observed for ASI score drug use*

	Improvement in ASI score drug use r
**Retention:**	**0.11**

*$p < 0.05$; **$p < 0.01$; ***$p < 0.001$

Marlowe et al. (2003)[48]

	Setting preferences (%)		
	Outpatient	Day treatment	No preference
Preference:	34	61	5

	Treatment outcomes		
	Matched	Not matched	Wilks Lamda, F
Maximum no. of consecutive Cocaine-free samples:			
Total no. of cocaine-free urines:			0.95, 0.56
Percentage of cocaine-free urines:			
Days of tenure in treatment:			

*$p < 0.05$; **$p < 0.01$; ***$p < 0.001$

McCrady et al. (2011)[49]

	(n) or (m, sd)	
	Individual	Couple
Preferences:	103	19
Attended sessions:	8.5 (4.4)	9.0 (4.2)

*$p < 0.05$; **$p < 0.01$; ***$p < 0.001$

McKay et al. (1995)[50]

(m, sd) or (%, n)

Months		Random		Nonrandom		ANCOVA		
		Day hospital (n=24)	Inpatient (n=24)	Day hospital (n=65)	Inpatient (n=31)	Day hospital vs. Inpatient	Random vs. Non-random	Inter-action
3	No. of drinking days	1.37 (4.59)	3.40 (7.23)	3.87 (7.63)	5.50 (9.86)	.09	.08	2.09
	Any days intoxicated	21.1 (4)	35.0 (7)	31.7 (19)	40.0 (8)	.75	2.36	1.53
	Any days of cocaine use	0.0 (0)	0.0 (0)	5.0 (3)	0.0 (0)			
	Treated in reha. again	5.3 (1)	15.8 (3)	6.7 (4)	10.0 (2)	.79	.14	.13
	Entered detoxifiction	5.3 (1)	0.0 (0)	0.0 (0)	0.0 (0)			
6	No. of drinking days	1.55 (3.14)	7.38 (10.19)	4.19 (7.20)	4.46 (8.30)			
	**Any days intoxicated**	20.0 (4)	57.1 (12)	39.7 (23)	29.2 (7)	1.37	.16	**10.04****
	Any days of cocaine use	0.0 (0)	23.8 (5)	1.7 (1)	4.2 (1)	1.72	3.03	2.41
	Treated in reha. again	10.0 (2)	9.5 (2)	13.8 (8)	12.5 (3)	.64	.87	.00
	Entered detoxifiction	5.0 (1)	4.8 (1)	1.7 (1)	4.2 (1)			
12	No. of drinking days	2.85 (5.19)	6.70 (9.05)	4.51 (7.65)	7.04 (9.00)			
	**Any days intoxicated**	20.0 (4)	55.0 (11)	35.1 (20)	39.1 (9)	0.06	.27	**4.13***
	Any days of cocaine use	10.0 (2)	20.0 (4)	1.8 (1)	4.3 (1)	2.46	2.97	.42
	Treated in reha. again	15.0 (3)	10.0 (2)	12.3 (7)	30.4 (7)	.08	1.19	2.21
	Entered detoxifiction	0.0 (0)	5.0 (1)	1.8 (1)	13.0 (3)			

*$p < 0.05$; **$p < 0.01$; ***$p < 0.001$

… continued on the next page

McKay et al. (1995)[50]

Months		(m, sd) or (%, n)						
		Random		Nonrandom		ANCOVA		
		Day hospital (n=24)	Inpatient (n=24)	Day hospital (n=65)	Inpatient (n=31)	Day hospital vs. Inpatient	Random vs. Non-random	Inter-action
3	No. of days paid in the previous 30 days	8.89 (10.08)	12.50 (10.33)	10.98 (10.59)	15.37 (10.22)	1.04	.13	.66
	**- paid on welfare**	21.1 (4)	25.0 (5)	20.0 (12)	0.0 (0)	.01	.58	**3.92***
	Any major conflicts: Family	10.5 (2)	10.0 (2)	15.0 (9)	20.0 (4)	.63	.84	.02
	Any major conflicts: Others	5.3 (1)	10.0 (2)	1.7 (1)	15.0 (3)	2.61	.06	.63
	With money from illegal activities	0.0 (0)	5.0 (1)	0.0 (0)	0.0 (0)			
	Who had been incarcerated	0.0 (0)	5.0 (1)	3.3 (2)	5.0 (1)			
6	No. of days paid in the previous 30 days	8.05 (9.29)	13.10 (11.18)	10.45 (11.01)	13.74 (10.48)			
	- paid on welfare	30.0 (6)	23.8 (5)	19.0 (11)	4.2 (1)	.02	1.24	1.17
	Any major conflicts: Family	10.0 (2)	4.8 (1)	13.8 (8)	16.7 (4)	.55	1.66	.67
	Any major conflicts: Others	10.0 (2)	9.5 (2)	6.9 (4)	12.5 (3)	.04	.20	.63
	With money from illegal activities	0.0 (0)	4.8 (1)	1.7 (1)	0.0 (0)			
	Who had been incarcerated	0.0 (0)	4.8 (1)	0.0 (0)	0.0 (0)			

… continued on the next page

*$p < 0.05$; **$p < 0.01$; ***$p < 0.001$

McKay et al. (1995)[50]

Months		(m, sd) or (%, n)						
		Random		Nonrandom		ANCOVA		
		Day hospital (n=24)	Inpatient (n=24)	Day hospital (n=65)	Inpatient (n=31)	Day hospital vs. Inpatient	Random vs. Non-random	Inter-action
12	No. of days paid in the previous 30 days	13.20 (9.98)	12.35 (9.83)	12.14 (10.27)	12.78 (10.67)			
	- paid on welfare	25.0 (5)	15.0 (3)	14.0 (8)	13.0 (3)	.31	.05	.84
	Any major conflicts: Family	5.0 (1)	5.0 (1)	17.5 (10)	8.7 (2)	.46	3.19	.42
	Any major conflicts: Others	10.0 (2)	5.0 (1)	7.0 (4)	8.7 (2)	.72	.00	.27
	With money from illegal activities	5.0 (1)	5.0 (1)	0.0 (0)	0.0 (0)			
	Who had been incarcerated	5.0 (1)	0.0 (0)	0.0 (0)	4.3 (1)			

$^{*}p < 0.05$; $^{**}p < 0.01$; $^{***}p < 0.001$

McKay et al. (1998)[51]

Months		(m, sd) or (%, sd)			
		Randomized		Self-Selected	
		Day hospital	Inpatient	Day hospital	Inpatient
3	Days of cocaine use	1.73 (4.59)	2.67 (4.94)	3.79 (7.91)	1.44 (3.55)
	Drug composite	0.08 (0.09)	0.11 (0.10)	0.11 (0.09)	0.13 (0.09)
	Days of alcohol use	2.96 (6.51)	2.65 (4.37)	1.88 (4.04)	1.50 (2.01)
	Alcohol composite	0.14 (0.24)	0.11 (0.16)	0.10 (0.18)	0.11 (0.18)
	Positive cocaine urine test	33 (13)	50 (17)	44 (11)	40 (4)
	Family-social composite	0.12 (0.18)	0.14 (0.20)	0.17 (0.20)	0.21 (0.27)
	Psychiatric composite	0.15 (0.23)	0.15 (0.22)	0.12 (0.19)	0.24 (0.25)
6	Days of cocaine use	1.91 (5.38)	3.38 (6.85)	2.35 (4.74)	2.76 (6.79)
	Drug composite	0.09 (0.10)	0.09 (0.10)	0.09 (0.09)	0.07 (0.12)
	Days of alcohol use	2.53 (6.46)	3.60 (5.60)	2.86 (6.50)	2.35 (3.66)
	Alcohol composite	0.08 (0.12)	0.12 (0.17)	0.06 (0.12)	0.03 (0.05)
	Positive cocaine urine test	43 (16)	39 (16)	57 (16)	60 (6)
	Family-social composite	0.12 (0.21)	0.16 (0.20)	0.11 (0.19)	0.11 (0.21)
	Psychiatric composite	0.10 (0.18)	0.12 (0.19)	0.05 (0.10)	0.04 (0.09)

… continued on the next page

*$p < 0.05$; **$p < 0.01$; ***$p < 0.001$

McKay et al. (1998)[51]

		(m, sd) or (%, sd)			
		Randomized		Self-Selected	
Months		Day hospital	Inpatient	Day hospital	Inpatient
12	Days of cocaine use	1.52 (4.18)	2.02 (4.00)	1.79 (3.84)	1.33 (3.12)
	Drug composite	0.06 (0.08)	0.09 (0.09)	0.07 (0.07)	0.07 (0.08)
	Days of alcohol use	3.17 (5.35)	3.88 (6.06)	4.33 (7.47)	1.94 (2.98)
	Alcohol composite	0.11 (0.17)	0.09 (0.15)	0.13 (0.16)	0.10 (0.14)
	Positive cocaine urine test	48 (19)	55 (18)	55 (12)	19 (3)
	Family-social composite	0.11 (0.15)	0.13 (0.19)	0.16 (0.21)	0.14 (0.21)
	Psychiatric composite	0.05 (0.12)	0.13 (0.18)	0.09 (0.14)	0.16 (0.27)

… continued on the next page

*$p < 0.05$; **$p < 0.01$; ***$p < 0.001$

McKay et al. (1998)[51]

Variable (n=171)	Setting (S)	Assign-ment (A)	Time (T)	SxA	SxT	AxT	SxAxT
	χ^2						
**Days cocaine**	0.41	2.49	**256.78*** **	0.31	3.34	**14.53** **	4.00
**Drug composite**	0.58	0.50	**341.97*** **	0.56	2.13	**13.99** **	0.98
**Days drinking**	0.51	2.39	**91.16*** **	1.10	1.07	2.47	0.93
**Alcohol composite**	0.42	0.27	**78.59*** **	0.02	1.23	1.34	1.78
**Family composite**	1.91	0.19	**55.30*** **	0.15	2.60	**8.15* **	0.57
**Psych. Composite**	1.51	0.30	**37.61*** **	0.06	**12.21** **	**12.84** **	**8.07* **

*p < 0.05; **p < 0.01; ***p < 0.001

Sterling et al. (1992)[52]

	(m, sd)					
	t0 (n=127)			t1 (n=87)		
**ASI**	Chose (n=67)	Assigned (n=60)	t/χ^2	Chose (n=51)	Assigned (n=36)	t/χ^2
Medical	0.22 (0.29)	0.24 (0.30)	0.47	0.21 (0.33)	0.19 (0.31)	0.28
Employment	0.89 (0.18)	0.82 (0.23)	1.72	0.90 (0.17)	0.82 (0.25)	1.66
**Alcohol**	0.12 (0.17)	0.20 (0.23)	**2.17***	0.13 (0.20)	0.12 (0.21)	0.22
Drug	0.20 (0.08)	0.20 (0.08)	0.47	0.10 (0.11)	0.11 (0.12)	0.46
Legal	0.09 (0.17)	0.12 (0.20)	0.93	0.04 (0.13)	0.09 (0.20)	1.26
Family	0.26 (0.24)	0.29 (0.20)	0.68	0.17 (0.20)	0.14 (0.20)	0.60
**Psychological**	0.33 (0.21)	0.24 (0.23)	**2.22****	0.28 (0.23)	0.27 (0.25)	0.21
**No. of days of cocaine use/30**	8.87 (9.98)	8.85 (9.39)	0.01	2.65 (5.39)	6.33 (9.59)	**2.28***
**Retention**						
Mean days				28.87 (34.51)	32.77 (29.81)	0.68

*$p < 0.05$; **$p < 0.01$; ***$p < 0.001$

Shared Decision Making

Joosten et al. (2008)[53]

Helping Alliance Questionnaire (HAQ) (m, sd)

			IG	CG	t, d
Patient	Interim	Cooperation:	20.1 (2.5)	19.7 (3.7)	0.73, 0.13
		Helpfulness:	20.1 (2.6)	20.2 (2.8)	-0.10, 0.04
		Total:	44.7 (4.1)	44.1 (6.3)	0.61, 0.11
	Exit	Cooperation:	20.5 (2.7)	21.2 (3.2)	-1.13, 0.24
		Helpfulness:	20.7 (2.3)	21.8 (3.0)	-0.14, 0.04
		Total:	46.6 (4.7)	47.5 (6.3)	-0.82, 0.16
Clinician	Interim	Cooperation:	18.9 (2.3)	18.6 (2.3)	0.63, 0.13
		**Helpfulness:**	**19.0 (2.4)**	**17.8 (3.2)**	**2.44*, 0.42**
		**Total:**	**42.2 (4.4)**	**40.2 (5.4)**	**2.01*, 0.41**
	Exit	Cooperation:	18.9 (2.3)	18.3 (2.5)	1.10, 0.25
		**Helpfulness:**	**19.5 (2.6)**	**17.6 (4.1)**	**2.82**, 0.55**
		**Total:**	**42.6 (4.2)**	**39.4 (6.0)**	**3.01**, 0.62**

… continued on the next page

*$p < 0.05$; **$p < 0.01$; ***$p < 0.001$

Joosten et al. (2008)[53]

Helping Alliance Questionnaire (HAQ) (m, sd)

			IG	CG	t
Patient - Clinician	Interim	Cooperation:	1.3 (2.8)	1.1 (3.8)	0.31
		Helpfulness:	1.4 (2.9)	2.5 (4.4)	-1.66
		Total:	2.9 (5.3)	3.9 (8.1)	-0.79
	Exit	Cooperation:	1.3 (2.9)	2.5 (3.4)	-1.70
		Helpfulness:	2.1 (2.9)	3.6 (4.2)	-1.88
		**Total:**	**3.7 (5.4)**	**7.0 (7.3)**	**-2.44 ***

*$p < 0.05$; **$p < 0.01$; ***$p < 0.001$

Joosten et al. (2009)[2]

	European Addiction Severity Index (Europe ASI) (m, sd)				
	t0		t1		F
	IG	CG	IG	CG	
Alcohol:	5.3 (2.5)	5.2 (2.0)	3.1 (2.4)	3.6 (2.5)	2.72
**Drugs:**	**3.3 (3.2)**	**3.2 (3.3)**	**1.6 (2.2)**	**2.3 (2.8)**	**7.40****
**Psychological problems:**	**5.8 (2.0)**	**5.8 (2.0)**	**3.0 (2.3)**	**4.0 (2.5)**	**5.91***
Physical health:	2.5 (2.0)	2.7 (2.2)	1.6 (1.9)	2.0 (2.1)	0.88
Family/ social relations:	4.0 (1.7)	4.5 (1.5)	2.0 (1.9)	2.7 (2.3)	0.55
Work/ education/ income:	3.4 (1.7)	3.5 (1.9)	2.7 (2.0)	2.7 (2.1)	0.01
Legal:	1.4 (1.8)	1.6 (1.9)	0.2 (0.7)	0.6 (1.4)	0.15
Gambling:	0.2 (0.9)	0.3 (1.1)	0.1 (0.7)	0.1 (0.5)	0.90

	EuroQoL-5D (EQ-5D) (m)				
	t0		t1		F
	IG	CG	IG	CG	
**EQ-5D utility score:**	**0.71**	**0.72**	**0.78**	**0.79**	**5.66****
**EQ-5D VAS score:**	**6.8**	**6.7**	**7.0**	**7.0**	**9.31****

**p* < 0.05; ***p* < 0.01; ****p* < 0.001

Joosten et al. (2010)[54]

Treatment goals alignment patient-clinician (r)

	Session 1	Session 2	Session 3
**Alcohol:**	**0.65****	**0.72****	**0.81****
**Drugs:**	**0.84****	**0.80****	**0.91****
**Psychological distress:**	**0.31****	**0.58****	**0.47****
**Psychotic symptoms:**	**0.20***	**0.53****	**0.73****
**Physical health:**	**0.28****	**0.44****	0.31
**Company:**	**0.34****	**0.49****	**0.50****
**Intimate relationships:**	**0.32****	**0.47****	**0.29**
**Childcare:**	**0.78****	**0.91****	**0.89****
**Education:**	0.05	0.08	**0.36***
**Daytime activities:**	**0.26***	0.27	**0.55****
**Accommodation:**	**0.78****	**0.82****	**0.80****
**Money:**	**0.52****	**0.60****	**0.45****

$^{*}p < 0.05$; $^{**}p < 0.01$; $^{***}p < 0.001$

Joosten et al. (2011)[55]

	Interpersonal Checklist- Revised patients (ICL-R) (m, sd)							
	t0		t1		t2		F	
	IG	CG	IG	CG	IG	CG	time	time/ condition
**PA:**	7.1 (3.0)	6.8 (3.3)	7.9 (2.7)	7.1 (2.8)	8.3 (2.8)	7.2 (3.0)	**4.93****	0.88
**BC:**	4.9 (2.7)	5.5 (2.8)	6.0 (2.7)	6.2 (2.7)	6.4 (2.8)	6.0 (2.5)	**8.44****	1.96
**DE:**	7.2 (3.1)	6.6 (2.5)	8.1 (2.5)	7.0 (2.2)	7.4 (2.5)	7.1 (2.5)	**3.61***	1.21
**FG:**	8.7 (3.1)	8.1 (2.9)	7.8 (3.2)	7.1 (2.6)	7.6 (3.2)	7.4 (2.9)	**8.38****	0.44
**nFnG:**	6.9 (3.0)	6.0 (2.9)	5.4 (2.5)	4.9 (2.6)	5.4 (3.0)	5.9 (2.9)	**13.17****	**4.31***
**HI:**	9.4 (4.1)	9.4 (3.4)	7.2 (3.5)	7.7 (3.5)	6.9 (3.7)	7.9 (3.5)	**26.92****	**1.37***
**JK:**	8.2 (3.9)	7.8 (2.3)	7.0 (2.3)	7.5 (2.1)	7.1 (3.2)	7.1 (3.1)	**4.98****	1.23
**LM:**	9.0 (2.6)	9.0 (2.7)	8.3 (2.4)	8.6 (2.5)	8.2 (2.5)	8.6 (2.4)	**3.73***	0.40
NO:	10.4 (3.2)	10.4 (3.9)	9.9 (2.5)	10.6 (2.7)	9.9 (2.7)	10.4 (2.8)	0.28	0.88
**nNnO:**	7.7 (2.8)	8.3 (3.2)	8.6 (2.3)	8.9 (2.6)	9.3 (2.3)	8.4 (2.7)	**7.12****	**5.01****
**Control:**	-3.3 (11.7)	-1.1 (11.2)	3.8 (9.5)	3.8 (10.8)	5.4 (12.0)	2.0 (11.1)	**32.02****	**5.44****
Affiliation:	6.3 (11.7)	7.1 (10.0)	4.4 (8.1)	7.5 (8.1)	5.3 (8.7)	7.8 (6.4)	0.67	1.19

(PA= managerial-autocratic; BC= narcissistic-competitive; DE= sadistic-aggressive; FG= rebellious-docile; nFnG= silent-reserved; HI= masochistic-self-efficacing; JK= dependent-docile; LM= cooperative-conventional; NO= hypernormal-responsible; nNnO= social-extravert; Control= control dimension; Affiliation= affiliation dimension)

… continued on the next page

*$p < 0.05$; **$p < 0.01$; ***$p < 0.001$

Joosten et al. (2011)[55]

Interpersonal Checklist- Revised clinicians (ICL-R) (m, sd)

	t0		t2		F (p)	
	IG	CG	IG	CG	time	time/ condition
PA:	6.7 (1.8)	7.1 (1.8)	6.8 (2.0)	7.8 (2.0)	2.66	1.31
BC:	6.3 (1.6)	5.0 (1.2)	6.3 (1.7)	5.4 (1.5)	0.70	0.70
**DE:**	6.6 (1.6)	6.5 (1.2)	6.8 (1.8)	7.4 (2.0)	**4.75***	2.10
FG:	5.1 (2.0)	5.7 (2.5)	4.9 (1.9)	5.2 (2.1)	0.65	0.13
nFnG:	3.3 (1.3)	3.4 (2.1)	3.5 (1.8)	2.7 (1.6)	0.76	3.77
HI:	5.5 (3.1)	5.3 (2.4)	5.1 (3.1)	4.3 (2.5)	3.47	0.62
JK:	6.0 (2.4)	6.7 (2.1)	5.9 (2.4)	6.4 (2.9)	0.33	0.07
LM:	7.0 (1.2)	7.7 (2.4)	6.9 (1.1)	7.6 (2.2)	0.04	0.00
**NO:**	9.1 (2.1)	10.0 (2.2)	8.2 (1.8)	8.9 (2.4)	**7.09***	0.05
nNnO:	9.0 (2.5)	9.7 (3.1)	9.3 (1.7)	9.5 (3.0)	0.02	0.37
**Control:**	8.3 (7.5)	8.1 (7.4)	8.6 (7.0)	10.6 (8.3)	**4.09***	2.51
Affiliation:	6.5 (6.5)	9.3 (7.8)	5.5 (5.3)	7.5 (8.0)	2.17	0.20

(PA= managerial-autocratic; BC= narcissistic-competitive; DE= sadistic-aggressive; FG= rebellious-docile; nFnG= silent-reserved; HI= masochistic-self-efficacing; JK= dependent-docile; LM= cooperative-conventional; NO= hypernormal-responsible; nNnO= social-extravert; Control= control dimension; Affiliation= affiliation dimension)

*$p < 0.05$; **$p < 0.01$; ***$p < 0.001$

Magura et al. (1988)[56]

	Positive urine samples (%)		
	T0	T1	Mean change
Program A (n= 50)	11.1	12.2	1.0
Program B (n= 64)	10.5	10.3	-0.3
Program C (n= 57)	22.0	27.1	5.1

	Client Attitudes towards Methadone Programs (CAMP) subscales					
	Program A n=41		Program B n=30		Program C n=50	
	T1	change	T1	change	T1	change
Fairness of rules:	2.54	-0.01	2.56	0.13	2.82	0.14
Attitude toward program doctor:	2.82	0.13	3.37	0.22	3.11	0.25
Attitude toward program nurse:	2.34	0.02	2.20	0.06	2.77	0.03
Attitude toward program couns.:	2.42	0.02	2.32	0.12	2.31	0.16
Attitude toward Meth. programs:	2.35	0.02	2.87	0.27	2.58	0.08
Input into treatment:	3.55	-0.06	3.40	-0.08	3.50	-0.01

… continued on the next page

*$p < 0.05$; **$p < 0.01$; ***$p < 0.001$

Magura et al. (1988)[56]

	Client Attitudes towards Methadone Programs (CAMP) subscales								
	Program A n=41			Program B n=30			Program C n=50		
	T0	T1	change	T0	T1	change	T0	T1	change
Rules:	2.55	2.54	-0.01	2.56	2.56	0.13	2.68	2.82	0.14
Doctor:	2.69	2.82	0.13	3.14	3.37	0.22	2.86	3.11	0.25
Nurses:	2.32	2.34	0.02	2.13	2.20	0.06	2.74	2.77	0.03
Counselors:	2.40	2.42	0.02	2.20	2.32	0.12	2.31	2.47	0.16
Opinion MMTP:	2.33	2.35	0.02	2.60	2.87	0.27	2.50	2.58	0.08
Input:	3.61	3.55	-0.06	3.48	3.40	-0.08	3.51	3.50	-0.01

(Rules= Fairness of rules; Doctor= Attitude toward program doctor; Nurses= Attitude toward program nurses; Counselors= Attitude toward program counselors; Opinion MMTP= Attitude toward Methadone programs; Input= Input into treatment)

$^{*}p < 0.05$; $^{**}p < 0.01$; $^{***}p < 0.001$

Neumann et al. (2006)[27]

		% or g/day (sd)		
		IG	CG	*p*
6 months	**At risk drinking:**	**21.7**	**30.4**	**
	Alcohol intake:	14 (6-26)	17 (6-32)	
	**Percent change from Baseline:**	**-35.7 (-73.4-0)**	**-20.5 (-66-0)**	*
12 months	At risk drinking:	37.3	42.6	
	**Alcohol intake:**	**21 (9-34)**	**23 (14-42)**	*
	**Percent change from Baseline:**	**-22.8 (-60 ±26)**	**-10.9 (-46.6 ±45.9)**	*

$^*p < 0.05$; $^{**}p < 0.01$; $^{***}p < 0.001$

… continued on the next page

Neumann et al. (2006)[27]

		Drinking status in %		
		IG	CG	p
6 months	Remained low-risk:	48.3	49.3	
	Change from low- to at-risk:	4.3	7.6	
	**Change from at- to low-risk:**	**30.0**	**20.3**	**
	Remained at-risk:	17.4	22.8	
12 months	Remained low-risk:	41.6	39.5	
	Change from low- to at-risk:	11.7	15.3	
	Change from at- to low-risk:	21.1	17.9	
	Remained at-risk:	25.6	27.3	

*$p < 0.05$; **$p < 0.01$; ***$p < 0.001$

Willemsen et al. (2006)[57]

		Smoking status in %			
		IG	CG	Difference	*p*
2 weeks	Quit attempt:	14.0	11.9	2.1 (2.0-2.1)	
	7-days abstinence:	8.6	6.4	2.2 (2.2-2.2)	
	Continuous abstinence:	-	-	-	
6 months	**Quit attempt**:	**31.0**	**22.0**	**8.8 (3.4-14.2)**	**
	**7-days abstinence:**	**20.2**	**13.6**	**6.6 (2.0-11.2)**	**
	Continuous abstinence:	5.0	5.1	0.1 (-1.9-2.1)	

Self-reported usage of treatments at 6 months follow-up	IG	CG	Difference
Any NRT:	13.5	7.9	5.6 (-1.7-9.9)
Bupropion:	7.1	10.5	3.4 (-2.6-9.4)
Any other category A method:	9.0	10.5	1.5 (-5.7-8.7)
Any category B method:	13.5	14.0	0.5 (-7.3-8.3)
Advice from family physician:	3.9	8.8	4.9 (-1.1-10.9)
No treatment (willpower only):	36.8	42.1	5.3 (-5.8-16.4)

… continued on the next page

*$p < 0.05$; **$p < 0.01$; ***$p < 0.001$

Willemsen et al. (2006)[57]

	Psychological outcomes in m (sd) or %				
	IG		CG		Difference
	T0	T1	T0	T1	
No. of treatments known:					
**Pharmacological (0-6)**	**2.7 (1.5)**	**3.7 (1.9)*****	**2.6 (1.4)**	**2.9 (1.3)*****	**IG > G*****
**Other cat. A (0-6)**	**0.9 (1.1)**	**2.3 (2.0)*****	**0.9 (1.0)**	**1.08 (1.1)*****	**IG > CG*****
**Cat. B (0-5)**	**1.6 (1.2)**	**2.4 (1.6)*****	1.6 (1.3)	1.7 (1.2)	**IG > G*****
**Attitude toward treatment:**	**0.28 (0.9)**	**0.47 (0.9)*****	0.27 (0.8)	0.29 (0.8)	**IG > CG****
General self-efficacy expect.:	-0.01 (1.1)	0.01 (1.0)	-0.15 (1.0)	-0.10 (1.0)	
**Expectation of success when using treatment:**	**0.74 (0.9)**	**0.95 (0.7)*****	0.76 (0.8)	0.73 (0.8)	**IG >CG*****
General intention to use treatment:	0.36 (1.2)	0.38 (1.2)	0.36 (1.2)	0.29 (1.2)	
Intention to use specific treatment:					
**Pharmacological**	27.5	30.8	**7.0**	**31.1***	
**Other cat. A**	**11.3**	**23.3*****	**15.9**	**22.6*****	
Cat. B	19.5	21.2	15.9	15.7	

$^{*}p < 0.05$; $^{**}p < 0.01$; $^{***}p < 0.001$
