# Supplementary material for: Patient Preferences and Shared Decision Making in the Treatment of Substance Use Disorders: A Systematic Review of the Literature
Source: PLoS One. 2016 Jan 5;11(1):e0145817. doi: 10.1371/journal.pone.0145817 (PMC4701396; doi:10.1371/journal.pone.0145817)
Supplement: S1 Table — (DOCX) [file pone.0145817.s002.docx]

**Search Terms**

| **Database** | **Keywords** | **Results** |
| --- | --- | --- |
| **Embase via Ovid** | (exp addiction/ or (exp drug abuse/ or drug abuse*.mp .or exp drug dependence/ or drug depend*.mp) or (medication abuse*.mp. or medication depend*.mp. not exp prescription drug/ or prescribe* drugs.mp.) or (substance related disorders*.mp. or alcohol abuse*.mp. or exp alcoholism/ or exp alcohol abuse/ or exp alcohol/ or alcohol depend*.mp.) AND (exp medical decision making/ or exp decision making) or (exp patient preference/ not exp paternalism/ or paternal* decis* mak*.mp.) AND (exp medical decision making/ or exp decision making or (shar* and decision* and mak*).mp. not exp paternalism/ or paternal* decis* mak*.mp.) or exp patient participation/ or patient particip*.mp. AND (Abstinence or percent days of abstinence).mp. or exp alcohol abstinence/ or exp abstinence/ or consume reduc*.mp. or exp “quality of life”/ or quality of life.mp. or exp patient satisfaction/ or patient satisfaction.mp. or exp patient compliance/ or patient adherence.mp. or treatment knowledge.mp. or decisional conflict.mp. or decisional quality.mp. or regret.mp.) NOT exp rat/ or rat*.mp. or exp mouse/ or mice.mp. | 296 |
| **Medline via Ovid** | (Exp addiction or (exp drug abuse/ or drug abuse*.mp. or exp drug dependence/ or drug depend*.mp. not exp prescription drug/ or prescribe* drugs.mp.) or (medication abuse*.mp. or medication depend*.mp. not exp prescription drug/ or prescribe* drugs.mp.) or (substance related disorders*.mp. or alcohol abuse*.mp. or exp alcoholism/ or exp alcohol abuse/ or exp alcohol/ or alcohol depend*.mp.) and (exp medical decision making/ or exp decision making/ or (shar* and decision* and mak*).mp. not exp paternalism/ or paternal* decis* mak*.mp.) or (exp patient preference/ or patient prefer*.mp.) or (exp patient participation/ or patient particip*.mp.) and (exp treatment planning or treatment*.mp. or exp drug therapy/ or exp methadone treatment/ or treatment match*.mp.) and ((Abstinence or per cent days of abstinence).mp. or exp alcohol abstinence/ or exp abstinence or consume reduc*.mp. or exp “quality of life”/ or quality of life.mp. or exp patient satisfaction/ or patient satisfaction.mp. or exp patient compliance/ or patient adherence.mp. or treatment knowledge.mp. or decisional conflict.mp. or decision quality.mp. or regret.mp.)) not (exp rat/ or rat*.mp. or exp mouse/ or mice.mp.) | 146 |

| **PsychInfo via Ovid** | (Exp addiction or (exp drug abuse/ or drug abuse*.mp. or exp drug dependence/ or drug depend*.mp. not exp prescription drug/ or prescribe* drugs.mp.) or (medication abuse*.mp. or medication depend*.mp. not exp prescription drug/ or prescribe* drugs.mp.) (substance related disorders*.mp. or alcohol abuse*.mp. or exp alcoholism/ or exp alcohol abuse/ or exp alcohol/ or alcohol depend*.mp.) and ((exp medical decision making/ or exp decision making/ or (shar* and decision* and mak*).mp. not exp paternalism/ or paternal* decis* mak*.mp.) or (patient prefer*.mp. or (patient and preference).mp.) or (exp patient participation/ or patient particip*.mp.)) )) and (exp treatment planning or treatment*.mp. or exp drug therapy/ or treatment match*.mp. or exp Drug Rehabilitation/) and ((Abstinence or percent days of abstinence).mp. or exp alcohol abstinence/ or exp abstinence/ or consume reduc*.mp. or exp “quality of life”/ or quality of life.mp. or exp patient satisfaction/ or patient satisfaction.mp. or exp patient compliance/ or patient adherence.mp. or treatment knowledge.mp. or decisional conflict.mp. or decision quality.mp. or regret.mp.) not (exp rat/ or rat*.mp. or exp mouse/ or mice.mp.) | 130 |
| --- | --- | --- |
| **Psyndex via Ovid** | (substanzabhaengigkeit.mp. or (substanz and abhaengigkeit) or alkoholabhaengigkeit.mp. or (alkohol and abhaengigkeit).mp. or alkoholmissbrauch.mp. or (alkohol and missbrauch).mp. or drogenabhaengigkeit.mp. or (drogen and abhaengigkeit).mp. or drogenmissbrauch.mp. or (drogen and missbrauch).mp. or medikamentenabhaengigkeit.mp. or (medikamenten and abhaengigkeit).mp.) and (behandlungsplanung.mp. or behandlung.mp. or behandlungszuweisung.mp.) and ((partizipative entscheidungsfindung.mp. or medizinische entscheidung.mp. or patientenpraeferenzen.mp. or (patienten and praeferenzen).mp. or shared decision-making.mp. or (shar* and decision* and mak*).mp. or patientenpartizipation.mp. or (patient and partizipation).mp. or patientenbeteiligung.mp. or (patient* and beteiligung).mp. or klientenbeteiligung or (klienten and beteiligung).mp. not paternalismus) and (abstinenz.mp. or konsumreduktion.mp. or (konsum and reduktion) .mp. or trinkreduktion.mp. or konsummuster.mp. or quality of life.mp. or lebensqualitaet.mp. or patientenzufriedenheit.mp. or (patient and zufriedenheit) .mp. or klientenzufriedenheit.mp. or (klienten and zufriedenheit) .mp. or (patienten and compliance) .mp. or (klienten and compliance) .mp. or (compliance or behandlung) .mp. or patient adherence.mp. or behandlungswissen.mp. or wissen.mp. or (behandlung and informationen) .mp. or entscheidungskonflikt.mp. or (entscheidung and konflikt) .mp. or entscheidungsqualitaet.mp. or (entscheidung and qualitaet) .mp. or bedauern.mp. or (entscheidung and bedauern) .mp.) not (ratten or maeuse or ratte or maus) | 7 |
|  | | **579** |

Electronic database searches were conducted on the 2^nd^ of July in 2013
