# Supplementary material for: Patient Preferences and Shared Decision Making in the Treatment of Substance Use Disorders: A Systematic Review of the Literature
Source: PLoS One. 2016 Jan 5;11(1):e0145817. doi: 10.1371/journal.pone.0145817 (PMC4701396; doi:10.1371/journal.pone.0145817)
Supplement: S3 Table — (DOCX) [file pone.0145817.s004.docx]

| **Excluded studies** | | |
| --- | --- | --- |
| **First author (year)** | **Reference** | **Reason for exclusion** |
| Adamson SJ (2001) | [Adamson SJ](http://www.motivationalinterviewing.org/bibliography?f%5bauthor%5d=4677), [Sellman DJ](http://www.motivationalinterviewing.org/bibliography?f%5bauthor%5d=4676). [Drinking goal selection and treatment outcome in out-patients with mild-moderate alcohol dependence](http://www.motivationalinterviewing.org/content/drinking-goal-selection-and-treatment-outcome-out-patients-mild-moderate-alcohol-dependence). Drug Alcohol Rev. 2001;20(4):351-359. | Informed choice |
| Adamson SJ (2010) | Adamson SJ, Heather N, Morton V, Raistrick D, Team UR. Initial preference for drinking goal in the treatment of alcohol problems: II. Treatment outcomes. Alcohol Alcohol. 2010;45(2):136-142. | Informed choice |
| Amass L (1996) | Amass L, Bickel WK, Crean JP, Higgins ST, Badger GJ. Preferences for clinic privileges, retail items and social activities in an outpatient buprenorphine treatment program. J Subst Abuse Treat. 1996;13(1):43-49. | Contingency management |
| Al-Otaiba Z(2008) | Al-Otaiba Z, Worden BL, McCrady BS, Epstein EE. Accounting for self-selected drinking goals in the assessment of treatment outcome. Psychol Addict Behav. 2008;22(3):439-443. | Informed choice |
| Alverson H (2000) | Alverson H, Alverson M, Drake RE. An ethnographic study of the longitudinal course of substance abuse among people with severe mental illness. Community Ment Health J. 2000;36(6):557-569. | No SDM / preferences |
| Anton RE (2005) | Anton RE, Randall CL. Measurement and choice of drinking outcome variables in the COMBINE Study. J Stud Alcohol – Suppl. 2005(15):104-109. | No SDM / preferences |
| Awgu E (2010) | Awgu E, Magura S, Rosenblum A. Heroin-dependent inmates' experiences with buprenorphine or methadone maintenance. J Psychoactive Drugs. 2010;42(3):339-346. | No SDM / preferences |
| Baker AL (2012) | Baker AL, Thornton LK, Hides L, Dunlop A. Treatment of cannabis use among people with psychotic disorders: a critical review of randomised controlled trials. Curr Pharm Des. 2012;18(32):4923-4937. | Severe mental illness, SUD comorbidity |
| Bammer G (2009) | Bammer G, Ritter A, Kutin JJ, Lintzeris N. Fast-tracking implementation through trial design: The case of buprenorphine treatment in Victoria. Aust NZ J Public Health. 2009;33(1):34-39. | No SDM/ preferences |
| Best D (2005) | Best D, Day E, Keaney F. The future for inpatient drug treatment services in England - The need to increase more than just capacity. J Subst Use. 2005;10(4):183-186. | No SDM / preferences |
| Bewick BM (2010) | Bewick BM, West R, Gill J, O'May F, Mulhern B, Barkham M, et al. Providing web-based feedback and social norms information to reduce student alcohol intake: a multisite investigation. J Med Internet Res. 2010;12(5):e59. | No SDM / preferences |
| Booth PG (1984) | Booth PG, Dale B, Ansari J. Problem drinkers' goal choice and treatment outcome: A preliminary study. Addict Behav. 1984;9(4):357-364. | Informed choice |
| Brener L (2009) | Brener L, Resnick I, Ellard J, Treloar C, Bryant J. Exploring the role of consumer participation in drug treatment. Drug Alcohol Depend. 2009;105(1-2):172-175. | Informed choice |
| Brown VB (2007) | Brown VB, Najavits LM, Cadiz S, Finkelstein N, Heckman JP, Rechberger E. Implementing an evidence-based practice: Seeking safety group. J Psychoactive Drugs. 2007;39(3):231-240. | No SDM / preferences |
| Brown CG (2005) | Brown CG, Stewart SH, Larsen SE. Experiences of harm reduction among women with alcohol use problems. Can J Community Ment Heal. 2005;24(1):95-113. | Informed choice |
| Bryant J (2008) | Bryant J, Bryant J, Saxton M, Madden A, Bath N, Robinson S. Consumer participation in the planning and delivery of drug treatment services: The current arrangements. Drug Alcohol Rev. 2008;27(2):130-137. | No preferences |
| Caetano R (1993) | Caetano R. Priorities for alcohol treatment research among US Hispanics. J Psychoactive drugs. 1993;25(1):53-60. | No data given |
| Cardellino JP (1997) | Cardellino JP. Decisional Balance Elements in the Natural Recovery/Reduction of Alcohol Consumption. Dissertation, Georgia State University; 1997. | No SDM / preferences |
| Carey KB (1999) | Carey KB, Purnine DM, Maisto SA, Carey MP, Barnes KL. Decisional balance regarding substance use among persons with schizophrenia. Community Ment Health J. 1999;35(4):289-299. | Motivational Interviewing |
| Carroll KM (2008) | Carroll KM, Ball SA, Martino S, Nich C, Babuscio TA, Nuro KF, et al. Computer-assisted delivery of cognitive-behavioral therapy for addiction: a randomized trial of CBT4CBT. Am J Psychiatry. 2008;165(7):881-888. | No SDM / preferences |
| Collett BJ (2002) | Collett BJ. The use of chronic opioid therapy for patients with nonmalignant pain. **Ann Longterm Care**. 2002;10(11):53-58. | Commentary |
| Collins SE (2005) | Collins SE. Weighing The Pros and Cons: Evaluating Decisional Balance as a Brief Motivational Intervention for At-risk College Drinkers. Addict Behav. 2005;30(7):1425-1430. | Motivational Interviewing |
| Costain WF (2008) | Costain WF. The effects of cannabis abuse on the symptoms of schizophrenia: patient perspectives. Int J Ment Health Nurs. 2008;17(4):227-235. | No SUD |
| Cunningham JA (1994) | Cunningham JA, Sobell LC, Sobell MB, Gaskin J. Alcohol and drug abusers' reasons for seeking treatment. Addict Behav. 1994;19(6):691-696. | Reasons for treatment |
| Dampz M (2012) | Dampz M, Falcato L, Beck T. Patientenzufriedenheit–Leuchtturm oder Irrlicht auf dem Weg zur Qualitätsbeurteilung ambulanter Suchtbehandlungen?. Abhängigkeiten. 2012;17(3):7-29. | No SDM / preferences |
| Davis S (1994) | Davis S. Drug treatment decisions of chemically-dependent women. Int J Addict. 1994;29(10):1287-1304. | No SDM / preferences |
| Davoli M (2003) | Davoli M, Perucci CA. Appropriateness of methadone maintenance treatment for opiate addiction: Treatment goals and effectiveness. Soz Praventivmed. 2003;48(Suppl. 1):S21-S22. | No SDM / preferences |
| Dawe S (1991) | Dawe S, Griffiths P, Gossop M, Strang J. Should opiate addicts be involved in controlling their own detoxification? A comparison of fixed versus negotiable schedules. Br J Addict. 1991;86(8):977-982. | Informed choice |
| DiClemente CC (2004) | DiClemente CC, Schlundt D, Gemmell L. Readiness and Stages of Change in Addiction Treatment. Am J Addict. 2004;13(2):103-119. | Motivational Interviewing |
| Dijkstra A (2008) | Dijkstra A, Jaspers M, van Zwieten M. Psychiatric and Psychological Factors in Patient Decision Making Concerning Antidepressant Use. J Consult Clinical Psychol. 2008;76(1):149-157. | No SUD |
| Donovan DM (2005) | Donovan DM. [Introduction to] Section 4: Assessment in Combined Pharmacotherapy and Behavior Therapy Trials of Alcoholism Treatment: Issues and Domains. J Stud Alcohol. 2005;Suppl. 15:92-93. | No SDM / preferences |
| Duffy SQ (2006) | Duffy SQ, Cowell AJ, Council CL, Shi W. Formal treatment, self-help, or no treatment for alcohol-use disorders? Evidence from the National Household Survey on Drug Abuse. J Stud Alcohol Drugs. 2006;67(3):363-372. | No SDM / preferences |
| Edlin BR (2002) | Edlin BR. Prevention and treatment of hepatitis C in injection drug users. Hepatology. 2002;36(5 SuppI 1):S210-S219. | No SDM / preferences |
| Finke J (1995) | Finke J, Teusch L, Gastpar M. Psychotherapie in der psychiatrischen Klinik--eine empirische Untersuchung über die Erwartungen der Patienten. Psychiatr Prax. 1995;22(3):112-116. | No SUD / preferences |
| Fischer EH (1995) | Fischer EH. Alcoholic patients' decisions about halfway houses What they say, what they do. J Subst Abuse Treat. 1996;13(2):159-164. | Preferences not described |
| Fleischmann H (2003) | Fleischmann H. Was erwarten psychisch Kranke von der Behandlung im psychiatrischen Krankenhaus. Psychiatr Prax. 2003;30 Suppl 2:S136-S139. | No SUD / preferences |
| Forys K (2007) | Forys K, McKellar J, Moos R. Participation in specific treatment components predicts alcohol-specific and general coping skills. Addict Behav. 2007;32(8):1669-1680. | No SDM / preferences |
| Foy DW (1979) | Foy DW, Rychtarik RG, O'Brien TP, Nunn LB. Goal choice of alcoholics: Effects of training controlled drinking skills. Behav Psychother. 1979;7(04):101-110. | Too old |
| Gitlow S (2008) | Gitlow S, Willenbring ML. Are medications that reduce risk of drinking or heavy drinking, or that promote abstinence, of value in the treatment of alcohol dependence?. Am J Addiction. 2008;17(1):1-5. | No SUD / preferences |
| Goldstein A (1975) | Goldstein A, Hansteen RW, Horns WH. Control of methadone dosage by patients. Jama-J Am Med Assoc. 1975;234(7):734-737. | Too old |
| Golub ET (2005) | Golub ET, Purvis LA, Sapun M, Safaeian M, Beyrer C, Vlahov D, et al. Changes in willingness to participate in HIV vaccine trials among HIV-negative injection drug users. AIDS Behav. 2005;9(3):301-309. | No SDM / preferences |
| Gray MT (2007) | Gray MT. Freedom and resistance: the phenomenal will in addiction. Nurs Philos. 2007;8(1):3-15. | No SDM / preferences |
| Hall SM (2007) | Hall SM, Gorecki JA, Reus VI, Humfleet GL, Munoz RF. Belief about drug assignment and abstinence in treatment of cigarette smoking using nortriptyline. Nicotine Tob Res. 2007;9(4):467-471. | No SDM / preferences |
| Heather N (2010) | Heather N, Adamson SJ, Raistrick D, Slegg GP, Team UR. Initial preference for drinking goal in the treatment of alcohol problems: I. Baseline differences between abstinence and non-abstinence groups. Alcohol Alcohol. 2010;45(2):128-135. | Informed choice |
| Hébert R (2009) | Hebert R. What's new in nicotine & tobacco research? Nicotine Tob Res. 2009;11(7):773-778. | No SDM / preferences |
| Hiltunen AJ (2002) | Hiltunen AJ, Eklund C. Withdrawal from methadone maintenance treatment. Reasons for not trying to quit methadone. Eur Addict Res. 2002;8(1):38-44. | Reasons for quitting |
| Hobbs BA (2009) | Hobbs BA, Elliott RL. When, and on what basis, do physicians decide to ignore a patient's choice in favor of administering a treatment involuntarily? J Med Assoc Ga. 2009;98(4):22-23. | No SUD |
| Hodgins DC (1997) | Hodgins DC, Leigh G, Milne R, Gerrish R. Drinking goal selection in behavioral self-management treatment of chronic alcoholics. Addict Behav. 1997;22(2):247-255. | Informed choice |
| Höppener PE (2013) | Hoppener PE, Godschalx-Dekker JA, van de Wetering BJM. Gedwongen opname bij stoornissen in of door het gebruik van middelen. Tijdschrift voor Psychiatrie. 2013;55(4):269-277. | No SDM / preferences |
| Holleran PR (1989) | Holleran PR, Novak AH. Support choices and abstinence in gay/lesbian and heterosexual alcoholics. Alcohol Treat Q. 1989;6(2):71-84. | No preferences |
| Jain R (1997) | Jain R, Thomasma DC. Discontinuing life support in an infant of a drug-addicted mother: whose decision is it? Camb Q Healthc Ethics. 1997;6(1):48-54 | Informed choice |
| Jakobsson A (2005) | Jakobsson A, Hensing G, Spak F. Developing a willingness to change: Treatment-seeking processes for people with alcohol problems. Alcohol Alcohol. 2005;40(2):118-123. | Motivational Interviewing |
| Kahler CW (2007) | Kahler CW, Daughters SB, Leventhal AM, Gwaltney CJ, Palfai TP. Implicit associations between smoking and social consequences among smokers in cessation treatment. Behav Res Ther. 2007;45(9):2066-2077. | No SDM / preferences |
| Kelly JF (2008) | Kelly JF, Brown SA, Abrantes A, Kahler CW, Myers M. Social recovery model: an 8-year investigation of adolescent 12-step group involvement following inpatient treatment. Alcohol Clin Exp Res. 2008;32(8):1468-1478. | Adolescents < 18 |
| Kelly SM (2012) | Kelly SM, Brown BS, Katz EC, O'Grady KE, Mitchell SG, King S, et al. A comparison of attitudes toward opioid agonist treatment among short-term buprenorphine patients. Am J Drug Alcohol Abuse. 2012;38(3):233-238. | Attitude |
| Kirby KC (2008) | Kirby KC, Kerwin ME, Carpenedo CM, Rosenwasser BJ, Gardner RS. Interdependent group contingency management for cocaine-dependent methadone maintenance patients. J Appl Behav Anal. 2008;41(4):579-595. | No SDM / preferences |
| Kolodziej ME (2012) | Kolodziej ME, Muchowski PM, Hamdi NR, Morrissette P, McGowan AJ, Weiss RD. Adaptation of the patient feedback survey at a community treatment setting. Am J Addict. 2012;21(1):63-71. | Evaluation measurement |
| Kuusisto K (2011) | Kuusisto K, Knuuttila V, Saarnio P. Pre-treatment expectations in clients: impact on retention and effectiveness in outpatient substance abuse treatment. Behav Cogn Psychoth. 2011;39(3):257-271. | No SDM / preferences |
| Laaksonen E (2011) | Laaksonen E, Lahti J, Sinclair JD, Heinälä P, Alho H. Predictors for the efficacy of naltrexone treatment in alcohol dependence: sweet preference. Alcohol Alcoholism. 2011;46(3):308-311. | No treatment or participation preference |
| LaFave LM (1999) | LaFave LM, Echols LD. An argument for choice. An alternative women's treatment program. J Subst Abuse Treat. 1999;16(4):345-352. | No SDM / preferences |
| Laudet AB (2009) | Laudet AB, Stanick V, Sands B. What could the program have done differently? A qualitative examination of reasons for leaving outpatient treatment. J Subst Abuse Treat. 2009;37(2):182-190. | No SDM / preferences |
| Lefebvre L (2010) | Lefebvre L, Midmer D, Boyd JA, Ordean A, Graves L, Kahan M, et al. Participant perception of an integrated program for substance abuse in pregnancy. J Obstet Gynaecol. 2010;39(1):46-52. | No SDM / preferences |
| Loneck BM (1989) | Loneck BM, Kola, LA. Using the conflict-theory model of decision making to predict outcome in the alcoholism intervention. Alcohol Treat Q. 1989;5(3-4):119-136. | Conflict Theory |
| Longabaugh R (2005) | Longabaugh R, Zweben A, LoCastro JS, Miller WR. Origins, issues and options in the development of the combined behavioral intervention. J Stud Alcohol. 2005;66(Suppl. 15):179-187. | No SDM / preferences |
| Loughland C (2010) | Loughland C, Draganic D, McCabe K, Richards J, Nasir A, Allen J, Catts S, Jablensky A, Henskens F, Michie P, Mowry B, Pantelis C, Schall U, Scott R, Tooney P, Carr V. Australian Schizophrenia Research Bank: a database of comprehensive clinical, endophenotypic and genetic data for aetiological studies of schizophrenia. Aust NZ J Psychiat. 2010;44(11):1029-1035. | No SUD |
| Luquiens A (2011) | Luquiens A, Reynaud M, Aubin HJ. Is controlled drinking an acceptable goal in the treatment of alcohol dependence? A survey of french alcohol specialists. Alcohol Alcohol. 2011;46(5):586-591. | Informed choice |
| Lynd LD (2010) | Lynd LD, Najafzadeh M, Colley L, Byrne MF, Willan AR, Sculpher MJ, et al. Using the incremental net benefit framework for quantitative benefit-risk analysis in regulatory decision-making--a case study of alosetron in irritable bowel syndrome. Value Health. 2010;13(4):411-417. | No SUD |
| Maddux JF (1995) | Maddux JF, Desmond DP, Vogtsberger KN. Patient‐Regulated Methadone Dose and Optional Counseling in Methadone Maintenance. AM J Addiction. 1995;4(1):18-32. | Informed Choice |
| Maddux JF (1997) | Maddux JF, Prihoda TJ, Vogtsberger KN. The relationship of methadone dose and other variables to outcomes of methadone maintenance. Am J Addict. 1997;6(3):246-255. | Informed Choice |
| Mathieu D (1995) | Mathieu D. Mandating treatment for pregnant substance abusers: a compromise. Polit Life Sci. 1995;14(2):199-208. | No SDM / preferences |
| McKinney KA (2010) | McKinney KA, Greenfield BG. Self-compliance at ‘Prozac campus’. Anthropol Med. 2010;17(2):173-185. | No SUD |
| McMahon J (1992) | McMahon J, Jones BT. The change process in alcoholics: client motivation and denial in the treatment of alcoholism within the context of contemporary nursing. J Adv Nurs. 1992;17(2):173-186. | Motivation |
| Melnick G (2008) | Melnick G, Wexler HK, Cleland CM. Client consensus on beliefs about abstinence: effects on substance abuse treatment outcomes. Drug Alcohol Depend. 2008;93(1-2):30-37. | Beliefs about abstinence |
| Moyers TB (2011) | Moyers TB, Houck J. Combining motivational interviewing with cognitive-behavioral treatments for substance abuse: Lessons from the COMBINE Research Project. Cog Behav Pract. 2011;18(1):38-45. | Motivational Interviewing |
| Nachega JB (2006) | Nachega JB, Knowlton AR, Deluca A, Schoeman JH, Watkinson L, Efron A, et al. Treatment supporter to improve adherence to antiretroviral therapy in HIV-infected South African adults: A qualitative study. J Acquir Immune Defic Syndr. 2006;43(Suppl. 1):S127-S133. | No SDM / preferences |
| Neale J (2013) | Neale J, Nettleton S, Pickering L. Does recovery-oriented treatment prompt heroin users prematurely into detoxification and abstinence programmes? Qualitative study. Drug Alcohol Depend. 2013;127(1-3):163-169. | No SDM / preferences |
| Nelle AC (2005) | Nelle AC. Solution and resource-oriented addiction treatment with the choices of abstinence or controlled drinking. J Fam Psychol. 2005;16(3):57-68. | No SDM / preferences |
| Neuner B (2007) | Neuner B, Dizner-Golab A, Gentilello L, Habrat B, Mayzner-Zawadzka E, Gorecki A, et al. Trauma Patients’ Desire for Autonomy in Medical Decision Making is Impaired by Smoking and Hazardous Alcohol Consumption – a Bi-national Study. J Int Med Res. 2007;35(5):609-614. | Cognitive impairment |
| O’Leary TA (2002) | O'Leary TA, Brown SA, Colby SM, Cronce JM, D'Amico EJ, Fader JS, Geisner IM, Larimer E, Maggs JL, McCrady B, Palmer S, Schulenberg J, Monti PM. Treating adolescents together or individually? Issues in adolescent substance abuse interventions. Alcohol Clin Exp Res. 2002;26(6):890-899. | Adolescents |
| Pachman JS (1978) | Pachman JS, Foy DW, Van Erd M. Goal choice of alcoholics: a comparison of those who choose total abstinence vs. those who choose responsible, controlled drinking. J Clin Psychol. 1978;34(3):781-783. | Too old |
| Peckham RH (1977) | Peckham RH. Uses of individualized client goals in the evaluation of drug and alcohol programs. Am J Drug Alcohol Ab. 1977;4(4):555-570. | Too old |
| Pérez de los Cobos J (2005) | Perez de Los Cobos J, Trujols J, Valderrama JC, Valero S, Puig T. Patient perspectives on methadone maintenance treatment in the Valencia Region: dose adjustment, participation in dosage regulation, and satisfaction with treatment. Drug Alcohol Depend. 2005;79(3):405-412. | Informed Choice |
| Perestelo-Perez L (2011) | Perestelo-Perez L, Gonzalez-Lorenzo M, Perez-Ramos J, Rivero-Santana A, Serrano-Aguilar P. Patient involvement and shared decision-making in mental health care. Curr Clin Pharmacol. 2011;6(2):83-90. | No SUD |
| Pratt TC (1977) | Pratt TC, Linn MW, Carmichael JS, Webb NL. The alcoholic's perception of the ward as a predictor of aftercare attendance. J Clin Psychol. 1977;33(3):915-918. | No SDM / preferences |
| Project MATCH (1999) | Project MATCH Research Group. Commentaries on Project MATCH: matching alcohol treatments to client heterogeneity. Addiction. 1999;94(1):31-69. | Commentary |
| Reisinger HS (2009) | Reisinger HS, Schwartz RP, Mitchell SG, Peterson JA, Kelly SM, O'Grady KE, et al. Premature discharge from methadone treatment: Patient perspectives. J Psychoactive Drugs. 2009;41(3):285-296. | No SDM / preferences |
| Robles E (2001) | Robles E, Miller FB, Gilmore-Thomas KK, McMillan DE. Implementation of a clinic policy of client-regulated methadone dosing. J Subst Abuse Treat. 2001;20(3):225-230. | Informed Choice |
| Rodler R (2006) | Rodler R, Hartel-Petri R, Steinmann J, Wolfersdorf M. Qualifizierte stationäre Drogenentzugsbehandlung. Anzahl und Dauer notwendiger Entgiftungsbehandlungen bei Drogenpatientlnnen vor Entwöhnungsbehandlungsantritt. Krankenhauspsychiatrie. 2006;17(1):15-18. | No SDM / preferences |
| Rohrer JE (1999) | Rohrer JE, Hilsenrath P. Client satisfaction with substance abuse treatment. Health Mark Q. 1999;17(2):31-42. | No SDM / preferences |
| Rohsenow DJ (2008) | Rohsenow DJ, Tidey JW, Miranda Jr R, McGeary JE, Swift RM, Hutchison KE, et al. Olanzapine Reduces Urge to Smoke and Nicotine Withdrawal Symptoms in Community Smokers. Exp Clin Psychopharm. 2008;16(3):215-222. | No SDM / preferences |
| Roose SP (2003) | Roose SP. Compliance: the impact of adverse events and tolerability on the physician's treatment decisions. Eur Neuropsychopharmacol. 2003;13 Suppl 3:S85-S92. | No SUD |
| Rowan PJ (2007) | Rowan PJ, Dunn NJ, El‐Serag HB, Kunik ME. Views of hepatitis C virus patients delayed from treatment for psychiatric reasons. J Viral Hepatitis. 2007;14(12):883-889. | No SUD |
| San L (1993) | San L, Tato J, Torrens M, Castillo C, Farré M, Camí J. Flunitrazepam consumption among heroin addicts admitted for in-patient detoxification. Drug Alcohol Depend. 1993;32(3):281-286. | No preference |
| Sanz Pozo B (2006) | Sanz Pozo B, Camarelles Guillem F, de Miguel Diez J.  Manejo de las recaidas en la deshabituacion tabaquica. Rev Clin Esp. 2006;206(3):150-152. | Not English |
| Schalast N (2000) | Schalast N. Zur Frage der Behandlungsmotivation bei Patienten des Massregelvollzugs gemass section 64 StGB. Psychiatr Prax. 2000;27(6):270-276. | No preferences / SDM |
| Selzer J (2012) | Selzer J, Stancliff S. Buprenorphine maintenance therapy in opioid-addicted health care professionals returning to clinical practice: A hidden controversy. Mayo Clin Proc. 2012;87(8):805-806. | No SDM |
| Singhal S (1992) | Singhal S, Nagalakshmi SV. Relapse in Alcoholism-Psychosocial Study. Nimhans J. 1992;10(1):47-49. | No preferences / SDM |
| Smyth BP (2006) | Smyth BP. Author's reply. Br J Psychiatry. 2006;188(3):292-293. | Commentary |
| Souza MM (2012) | Souza YMM. La alianza terapeutica y el apego al tratamiento: Metodologia tecnica en adicciones. Rev Mex Neuroci. 2012;13(6):324-330. | No SDM / preferences |
| Sowers W (2005) | Sowers W. Transforming systems of care: The American Association of Community Psychiatrists Guidelines for Recovery Oriented Services. Community Ment Health J. 2005;41(6):757-774. | No data given |
| Srisurapanont M (2002) | Srisurapanont M*,* Jarusuraisin N*.* Opioid antagonists for alcohol dependence*.* Cochrane Database Syst Rev*.* 2002;(2):CD001867. | No SDM / preferences |
| Steinkopf L (2012) | Steinkopf L. Enhancing drug compliance and the placebo effect by raising subjective expectations. Med Hypotheses. 2012;79(5):698-700. | No SUD |
| Storbjörk J (2012) | Storbjörk J. On the significance of social control: Treatment-entry pressures, self-choice and alcohol and drug dependence criteria one year after treatment. Int J Soc Wel. 2012;21(2):160–173. | Informed choice |
| Sylvestre DL (2005) | Sylvestre DL. Treating hepatitis C virus infection in active substance users. Clin Infect Dis. 2005;40(Suppl. 5):S321-S324. | No SDM / preferences |
| Tacke U (2009) | Tacke U, Uosukainen H, Kananen M, Kontra K, Pentikanen H. A pilot study about the feasibility and cost-effectiveness of electronic compliance monitoring in substitution treatment with buprenorphine-naloxone combination. Journal of Opioid Management. 2009;5(6):321-329. | No SDM / preferences |
| Tetzlaff BT (2005) | Tetzlaff BT, Kahn JH, Godley SH, Godley MD, Diamond GS, Funk RR. Working alliance, treatment satisfaction, and patterns of posttreatment use among adolescent substance users. Psychol Addict Behav. 2005;19(2):199-207. | Adolescents |
| Tibaldi G (2011) | Tibaldi G, Salvador-Carulla L, Garcia-Gutierrez JC. From treatment adherence to advanced shared decision making: New professional strategies and attitudes in mental health care. Curr Clin Pharmacol. 2011;6(2):91-99. | No SUD |
| Timko C (1993) | Timko C, Finney JW, Moos RH, Moos BS, Steinbaum DP. The process of treatment selection among previously untreated help-seeking problem drinkers. J Subst Abuse.1993;5(3):203-220. | No preferences |
| Trujols J (2012) | Trujols J, Garijo I, Sinol N, del Pozo J, Portella MJ, Perez de los Cobos J. Patient satisfaction with methadone maintenance treatment: the relevance of participation in treatment and social functioning. Drug Alcohol Depend. 2012;123(1-3):41-47. | Severe mental illness, SUD comorbidity |
| Tsai J (2010) | Tsai J, Bond GR, Salyers MP, Godfrey JL, Davis KE. Housing preferences and choices among adults with mental illness and substance use disorders: a qualitative study. Community Ment Health J. 2010;46(4):381-388. | Severe mental illness, SUD comorbidity |
| Vabret F (2006) | Vabret F. Modalites du sevrage alcoolique. Rev Prat. 2006;56(10):1093-1099. | No SDM / preferences |
| VanDeMark NR (2010) | VanDeMark NR, Burrell NR, Lamendola WF, Hoich CA, Berg NP, Medina E. An exploratory study of engagement in a technology-supported substance abuse intervention. Subst Abuse Treat Prev. 2010;5-10. | Motivational Interviewing |
| Vanderplasschen W (2013) | Vanderplasschen W, Colpaert K, Autrique M, Rapp RC, Pearce S, Broekaert E, et al. Therapeutic communities for addictions: A review of their effectiveness from a recovery-oriented perspective. Sci World J. 2013;2013:427817. | No SDM / preferences |
| Vardy J (2012) | Vardy J, Pond G, Dodd A, Warr D, Seruga B, Clemons M, et al. A randomized double-blind placebo-controlled cross-over trial of the impact on quality of life of continuing dexamethasone beyond 24h following adjuvant chemotherapy for breast cancer. Breast Cancer Res Treat. 2012;136(1):143-151. | No SUD |
| Wagner GL (2005) | Wagner GJ, Ryan GW. Hepatitis C virus treatment decision-making in the context of HIV co-infection: the role of medical, behavioral and mental health factors in assessing treatment readiness. AIDS. 2005;19 Suppl 3:190-198. | No SUD |
| Wallace AS (2009) | Wallace AS, Freburger JK, Darter JD, Jackman AM, Carey TS. Comfortably numb? Exploring satisfaction with chronic back pain visits. Spine J. 2009;9(9):721-728. | No SUD |
| Wiggers LCW (2005) | Wiggers LCW, Stalmeier PFM, Oort FJ, Smets EMA, Legemate DA, de Haes JCJM. Do patients' preferences predict smoking cessation? Prev Med. 2005;41(2):667-675. | No SDM / preferences |
