# Supplementary material for: Patient Preferences and Shared Decision Making in the Treatment of Substance Use Disorders: A Systematic Review of the Literature
Source: PLoS One. 2016 Jan 5;11(1):e0145817. doi: 10.1371/journal.pone.0145817 (PMC4701396; doi:10.1371/journal.pone.0145817)
Supplement: S4 Table — (DOCX) [file pone.0145817.s005.docx]

# Results of MMAT

| *Box* | *Methodological quality criteria* |  | | | | | | | | |  | | *Study IDs/Rating scores* | | | | | | | | | | | | | | | | | | |
| --- | --- | --- | --- | --- | --- | --- | --- | --- | --- | --- | --- | --- | --- | --- | --- | --- | --- | --- | --- | --- | --- | --- | --- | --- | --- | --- | --- | --- | --- | --- | --- |
| **A** | **Screening questions** | **16** | **26** | **28** | **5** | **19** | **9** | **7** | **11** | **18** | | **4** | | **1** | **2** | **3** | **6** | **8** | **10** | **12** | **13** | **14** | **15** | **17** | **20** | **22** | **21** | **23** | **24** | **25** | **27** |
| 1 | Clear qualitative and quantitative research or mixed method question? | ✓ | ✓ | ✓ | ✓ | ✓ | ✓ | ✓ | ✓ | ✓ | | ✓ | | ✓ | ✓ | ✓ | ✓ | ✓ | ✓ | ✓ | ✓ | ✓ | ✓ | ✓ | ✓ | ✓ | ✓ | ✓ | ✓ | ✓ | ✓ |
| 2 | Do the collected data allow address the research question? | 🗶 | ✓ | ✓ | ✓ | ✓ | ✓ | ✓ | ✓ | ✓ | | ✓ | | ✓ | ✓ | ✓ | ✓ | 🗶 | ✓ | n/a | ✓ | ✓ | ✓ | ✓ | ✓ | ✓ | ✓ | ✓ | ✓ | ✓ | ✓ |
| **B** | **Quantitative randomized controlled (trials)** | **16** | **26** | **28** | **5** | **19** | **9** | **7** | **11** | **18** | | **4** | | **1** | **2** | **3** | **6** | **8** | **10** | **12** | **13** | **14** | **15** | **17** | **20** | **22** | **21** | **23** | **24** | **25** | **27** |
| 1 | Clear description of the randomization? |  |  |  |  |  |  |  |  |  | |  | | 🗶 |  | ✓ |  | 🗶 |  | 🗶 | 🗶 |  |  |  | ✓ | ✓ |  | ✓ |  | 🗶 | 🗶 |
| 2 | Clear description of the allocation concealment? |  |  |  |  |  |  |  |  |  | |  | | 🗶 |  | 🗶 |  | 🗶 |  | 🗶 | 🗶 |  |  |  | ✓ | ✓ |  | ✓ |  | 🗶 | 🗶 |
| 3 | Complete outcome data? |  |  |  |  |  |  |  |  |  | |  | | ✓ |  | 🗶 |  | ✓ |  | ✓ | ✓ |  |  |  | 🗶 | ✓ |  | 🗶 |  | ✓ | ✓ |
| 4 | Low withdrawal/drop-out? |  |  |  |  |  |  |  |  |  | |  | | ✓ |  | 🗶 |  | 🗶 |  | ✓ | ✓ |  |  |  | 🗶 | 🗶 |  | 🗶 |  | 🗶 | 🗶 |
|  |  |  |  |  |  |  |  |  |  |  | |  | |  |  |  |  |  |  |  |  |  |  |  |  |  |  |  |  |  |  |
| **C** | **Quantitative non-randomized** | **16** | **26** | **28** | **5** | **19** | **9** | **7** | **11** | **18** | | **4** | | **1** | **2** | **3** | **6** | **8** | **10** | **12** | **13** | **14** | **15** | **17** | **20** | **22** | **21** | **23** | **24** | **25** | **27** |
| 1 | Are participants recruited in a way that minimizes selection bias? |  |  |  | ✓ |  |  |  |  |  | |  | |  | 🗶 |  | ✓ |  |  |  |  | ✓ | ✓ | ✓ |  |  | ✓ |  | 🗶 |  |  |
| 2 | Are measurements appropriate regarding the exposure/intervention and outcomes? |  |  |  | ✓ |  |  |  |  |  | |  | |  | ✓ |  | 🗶 |  |  |  |  | ✓ | ✓ | ✓ |  |  | ✓ |  | 🗶 |  |  |
| 3 | In groups being compared, are the participants comparable, or do researchers take into account the differences between these groups? |  |  |  | 🗶 |  |  |  |  |  | |  | |  | 🗶 |  | 🗶 |  |  |  |  | ✓ | ✓ | ✓ |  |  | 🗶 |  | 🗶 |  |  |
| 4 | Are there complete outcome data, and, when applicable, an acceptable response rate, or an acceptable follow-up rate for cohort studies? |  |  |  | ✓ |  |  |  |  |  | |  | |  | 🗶 |  | 🗶 |  |  |  |  | ✓ | ✓ | 🗶 |  |  | ✓ |  | 🗶 |  |  |

| **D** | **Quantitative descriptive** | **16** | **26** | **28** | **5** | **19** | **9** | **7** | **11** | **18** | **4** | **1** | **2** | **3** | **6** | **8** | **10** | **12** | **13** | **14** | **15** | **17** | **20** | **22** | **21** | **23** | **24** | **25** | **27** |
| --- | --- | --- | --- | --- | --- | --- | --- | --- | --- | --- | --- | --- | --- | --- | --- | --- | --- | --- | --- | --- | --- | --- | --- | --- | --- | --- | --- | --- | --- |
| 1 | Is the sampling strategy relevant to address the quantitative research question? | 🗶 | ✓ | 🗶 |  | 🗶 | 🗶 | 🗶 | 🗶 | 🗶 | ✓ |  |  |  |  |  | ✓ |  |  |  |  |  |  |  |  |  |  |  |  |
| 2 | Is the sample representative of the population understudy? | 🗶 | ✓ | 🗶 |  | ✓ | ✓ | ✓ | ✓ | 🗶 | 🗶 |  |  |  |  |  | ✓ |  |  |  |  |  |  |  |  |  |  |  |  |
| 3 | Are measurements appropriate? | 🗶 | ✓ | ✓ |  | 🗶 | ✓ | 🗶 | 🗶 | ✓ | ✓ |  |  |  |  |  | ✓ |  |  |  |  |  |  |  |  |  |  |  |  |
| 4 | Is there an acceptable response rate? | ✓ | ✓ | ✓ |  | ✓ | 🗶 | ✓ | ✓ | ✓ | 🗶 |  |  |  |  |  | ✓ |  |  |  |  |  |  |  |  |  |  |  |  |
| **E** | **Total score** | ***** | ******** | ** | ******* | ****** | ****** | ****** | ****** | ****** | ****** | ****** | ***** | ***** | ***** | ***** | ******** | ****** | ****** | ******** | ******** | ******* | ****** | ******* | ******* | ****** | **/** | ***** | ***** |

16^[32]^; 26^[33]^; 28^[34]^; 5^[35]^; 19^[36]^; 9^[37]^; 7^[38]^; 11^[39]^; 18^[40]^; 4^[41]^; 1^[42]^; 2^[43]^; 3^[44]^; 6^[45]^; 8^[46]^; 10^[47]^; 12^[48]^; 13^[49]^; 14^[50]^; 15^[51]^; 17^[52]^; 20^[53]^; 22^[2]^; 21^[54]^; 23^[55]^; 24^[56]^; 25^[27]^; 27^[57]^

Space = MMAT rating not applicable; n/a = not applicable; **🗶**MMAT criteria not described; **✓** MMAT criteria described

/ = Met 0% of MMAT criteria; * Met 25% of MMAT criteria; ** Met 50% of MMAT criteria; *** Met 75% of MMAT criteria; **** Met 100% of MMAT criteria
